# YOLO-LitchiVar: a lightweight and high-precision detection model for fine-grained litchi variety identification

**DOI:** 10.3389/fpls.2025.1677854

**Published:** 2026-01-29

**Authors:** Bing Xu, Xianjun Wu, Xueping Su, Wende Ke

**Affiliations:** 1 School of Computer, Guangdong University of Petrochemical Technology, Maoming, Guangdong, China; 2 Department of Mechanical and Energy Engineering, Southern University of Science and Technology, Shenzhen, China

**Keywords:** litchi variety classification, Lightweight model, YOLOv12, cross-level feature fusion, multi-scale feature alignment

## Abstract

Pengju Ren, Shanghai Maritime University, ChinaLitchi is a popular subtropical fruit with approximately 100 known varieties worldwide. Traditional post-harvest litchi variety classification primarily relies on manual identification, which suffers from low efficiency, strong subjectivity, and a lack of standardized systems. This study constructs an image dataset comprising the 12 most common litchi varieties found in commercial markets and proposes YOLO-LitchiVar, a lightweight and high-precision detection model that synergistically optimizes both computational efficiency and recognition accuracy for fine-grained litchi variety classification. The proposed model is built upon the YOLOv12 architecture and achieves significant performance improvements through the synergistic optimization of three novel modules. First, we introduce the DSC3k2 module to address lightweight design requirements, employing a depthwise separable convolutional structure that decouples standard convolution into spatial filtering and channel fusion. This innovation significantly reduces model complexity, decreasing parameters from 2.57 million to 2.20 million (a 14.1% reduction) and computational cost from 6.5G to 5.6G FLOPs (a 13.8% reduction). Second, we develop the C2PSA cross-layer feature aggregation module to enhance feature representation through multi-scale feature alignment and fusion, specifically improving shallow microtexture characterization capability. This module effectively addresses the missed detection problem of Icy-Flesh Litchi caused by the loss of micro-concave texture, increasing the recall rate from 0.492 to 0.706 (a 43.5% enhancement). Finally, we integrate an ECA attention mechanism to optimize discriminative performance by dynamically calibrating channel weights through adaptive kernel 1D convolution, thereby suppressing background noise (e.g., illumination variations) and features shared by similar varieties. This integration lowers the misclassification rate between Icy-Flesh Litchi and Osmanthus-Fragr Litchi from 0.462 to 0.340 (a 19.1% reduction). Experiments on a dataset of 11,998 multi-variety litchi images demonstrate that the YOLO-LitchiVar model achieves excellent comprehensive performance, with a mAP50–95 of 94.4%, which is 0.8% higher than the YOLOv12 baseline model. It also maintains lightweight advantages with a parameter count of 2.20 million and a computation volume of 5.6G FLOPs, making it suitable for mobile deployment. This study provides an efficient and effective solution for intelligent litchi variety identification with global applicability.

## 1 Introduction

Litchi is an important tropical and subtropical fruit crop known for its juicy flesh and distinctively rich, sweet flavor. Due to its specific cooling requirements for bud differentiation and sensitivity to low-temperature stress, global commercial cultivation of litchi is mainly concentrated within a narrow climatic zone between 17° and 26° north and south latitudes. Among the 16 major litchi-producing countries worldwide, China accounts for more than 70% of total production, occupying an absolutely dominant position. India, Vietnam, Thailand, and other countries are also important litchi producers, playing a key role in the supply of the international market.

There are many varieties of litchi, and their market values and price fluctuations vary considerably. For example, the unit prices of premium varieties such as “Osmanthus-Fragr Litchi” and “Glu-Rice Ciba Litchi” can be two to three times higher than those of common varieties. This price difference directly reflects the decisive influence of varietal characteristics on the industry’s value. Therefore, accurate identification of the main litchi varieties is of great industrial significance—it not only underpins the “quality-based pricing” mechanism but also promotes precise market alignment and industrial upgrading based on varietal characteristics. Clear variety labeling enables the market to avoid “second-best” substitutions and enhances consumer trust.

Currently, a major challenge in post-harvest treatment and market circulation of litchi is the lack of an efficient, objective, and standardized variety identification system. Traditional methods mainly rely on manual experience, using visual assessment of skin color, shape, pericarp cracking patterns, and taste for identification. These approaches are subjective, inefficient, difficult to standardize, and susceptible to operator fatigue, making them unsuitable for large-scale, rapid processing. This bottleneck severely restricts branding development and the realization of quality-based pricing within the litchi industry. Therefore, establishing a scientific, efficient, and standardized method for litchi variety identification is crucial for safeguarding product value, strengthening consumer confidence, and promoting industrial upgrading.

The YOLO (You Only Look Once) series of models have been widely applied in object detection tasks due to their high accuracy and rapid inference capability. However, when applied to the fine-grained task of litchi variety identification, existing YOLO models still exhibit limitations in lightweight design, shallow feature utilization, and precise discrimination among morphologically similar varieties. These shortcomings hinder deployment in resource-constrained mobile scenarios and limit accuracy in real-world applications.

To bridge these gaps, this study was motivated by the urgent need for an efficient, accurate, and standardized method to replace subjective manual identification in the litchi industry. We aim to develop a highly optimized model that achieves an optimal balance between recognition accuracy and computational efficiency, enabling real-time, in-field variety identification.

Based on the YOLOv12 architecture, this study proposes a progressive three-module co-optimization model, YOLO-LitchiVar, to achieve high-precision and low-latency variety discrimination. The main contributions of this work are summarized as follows:

- A novel lightweight backbone design: The DSC3k2 module replaces standard convolutions with a depthwise separable structure, reducing the model’s parameter count by 14.1% (from 2.57 million to 2.20 million) and computational cost by 13.8% (from 6.5 GFLOPs to 5.6 GFLOPs), thereby facilitating deployment on mobile devices.
- Enhanced feature representation for fine-grained discrimination: The C2PSA cross-layer feature aggregation module effectively aligns and fuses multi-scale features, enhancing the model’s capability to capture shallow microtextures (e.g., the micro-concave patterns of “Icy-Flesh Litchi”). This improvement addresses missed detections and increases the recall rate for challenging varieties by 43.5%.
- Improved discriminative performance under noise and similarity: The Efficient Channel Attention (ECA) mechanism dynamically calibrates channel-wise feature responses, effectively suppressing irrelevant background noise (e.g., illumination variations) and non-discriminative shared features, reducing the misclassification rate between “Icy-Flesh Litchi” and “Osmanthus-Fragr Litchi” by 19.1%.
- A comprehensive, publicly available dataset: A dataset comprising 11,998 images of 12 common commercial litchi varieties was established under natural lighting conditions in a controlled laboratory environment. This dataset provides a valuable benchmark for future agricultural image recognition research.

Through the synergistic optimization of these three core modules, the proposed YOLO-LitchiVar model surpasses the YOLOv12 baseline and other YOLO variants in key performance metrics while maintaining minimal computational overhead. This study provides an effective and scalable technological solution for intelligent, mobile-based litchi variety detection.

The remainder of this paper is organized as follows: Section 2 reviews related work on lightweight models and agricultural vision systems; Section 3 details the proposed methodology and YOLO-LitchiVar architecture; Section 4 describes the dataset and experimental setup; Section 5 presents and discusses the results of ablation and comparative experiments; and Section 6 concludes the paper and suggests directions for future research.

## 2 Related work

The integration of artificial intelligence (AI) into agricultural practices has catalyzed a transformative shift toward automated and intelligent farming systems. Leveraging powerful technologies such as machine learning (ML), computer vision (CV), and advanced data analytics, AI-enabled systems can achieve rapid and accurate recognition of biological features, significantly enhance operational efficiency through automation, and substantially increase the economic value of agricultural products by ensuring quality and standardization (Chamara et al., 2023). The application spectrum of visual inspection in agriculture is broad, encompassing critical tasks such as crop and disease classification (Chamara et al., 2023; Sharma et al., 2024), image segmentation (Chamara et al., 2023), object detection (Rong et al., 2019; Jiao et al., 2020; Hernández et al., 2021; Wang et al., 2022; Hu et al., 2024; Wu et al., 2024; Li et al., 2025), and counting (Chamara et al., 2023).

Substantial research efforts have been dedicated to applying deep learning to diverse agricultural challenges. Chamara et al (Chamara et al., 2023). developed the AICropCAM system, an edge-computing solution that deploys deep learning models for classification, segmentation, detection, and counting tasks, achieving a notable 91.26% accuracy in crop classification. Sharma et al (Sharma et al., 2024). demonstrated the efficacy of optimized ML architectures, including Inception V3 and EfficientNet, achieving a 97% accuracy rate for classifying diseases in crops such as cassava and maize. Beyond classification, Ghaderi Zefrehi et al (Zefrehi, 2024). employed a fusion strategy across multiple color spaces combined with a weighted voting mechanism to enhance the classification accuracy of potato foliar diseases. For object detection, Jiao et al (Jiao et al., 2020). proposed the AF-RCNN model, which uses an anchor-free region proposal network to address the challenge of detecting agricultural pests. Similarly, Rong et al (Rong et al., 2019). designed a dual-CNN architecture for detecting foreign objects in walnuts, reporting significant improvements in detection efficiency. In more complex scenarios involving multi-scale targets and category imbalances, Li et al (Li et al., 2025). introduced PD-YOLO, which incorporates multi-scale feature fusion and a dynamic detection head to improve the mean average precision (mAP) for weed detection in complex environments by 1.7%. Wang et al (Wang et al., 2022). designed the DSE-YOLO model with a detail semantic enhancement module to address category imbalance in multi-stage strawberry detection, achieving a high mAP. Furthermore, Hu et al (Hu et al., 2024). developed a model combining Vision Transformer and CNN dual-branch structures for cotton weed identification, reducing parameters by 1.52 × 10^6^ while maintaining accuracy. For small-target detection, Wu et al (Wu et al., 2024). proposed a multi-scale YOLO model for detecting citrus pests, achieving state-of-the-art performance through multi-scale feature extraction and a novel loss function. Collectively, these studies validate the potential of AI technologies in revolutionizing agricultural practices while highlighting persistent challenges such as data scarcity, variability in imaging quality, and the need for improved model generalization in unstructured environments (Rong et al., 2019; Jiao et al., 2020; Wang et al., 2022; Chamara et al., 2023; Hu et al., 2024; Sharma et al., 2024; Wu et al., 2024; Li et al., 2025).

The YOLO (You Only Look Once) series of models represents a cornerstone in the evolution of real-time object detection frameworks, renowned for their exceptional speed and accuracy. The architecture of YOLOv12, which serves as the baseline for this study, incorporates several innovations over its predecessors, including an advanced backbone network (R-ELAN) and an efficient area attention mechanism (A2) (Tian et al., 2024; Rabbani Alif and Hussain, 2025). The applicability of recent YOLO versions extends far beyond general object detection into specialized domains. In marine conservation, YOLOv12 has been used for real-time detection of marine litter, a crucial capability for supporting pollution mitigation efforts (Ma et al., 2025). In challenging underwater environments characterized by light attenuation and turbidity, YOLOv12 has been enhanced with physics-informed augmentation techniques to improve target detection (Nguyen, 2025). Within the agricultural sector, studies have explored its potential for crop protection (Rabbani Alif and Hussain, 2025) and fruit detection (RF-DETR object detection vs YOLOv12: A study of transformer-based and CNN-based architectures for single-class and multi-class greenfruit detection in complex orchard environments under label ambiguity, 2025). For instance, Sapkota et al (RF-DETR object detection vs YOLOv12: A study of transformer-based and CNN-based architectures for single-class and multi-class greenfruit detection in complex orchard environments under label ambiguity, 2025). conducted a comparative analysis between RF-DETR (a transformer-based model) and YOLOv12 for detecting green fruits in complex orchard environments, evaluating robustness under realistic conditions such as label blurring, occlusion, and background fusion. YOLO models have also been applied to waste management for real-time garbage detection and classification (Dipo et al., 2025) and to industrial quality control for printed circuit board (PCB) defect detection through lightweight architectures such as MAS-YOLO (Yin et al., 2025). This demonstrated versatility across diverse fields highlights the robustness and adaptability of the YOLO architecture. However, deploying these models for fine-grained agricultural classification tasks, such as distinguishing between visually similar litchi varieties, presents unique challenges. These include the need for extreme model lightweighting without compromising accuracy, the effective exploitation of shallow features for microtexture recognition, and enhanced discrimination capabilities to overcome interclass similarities—gaps not fully addressed by existing generic models.

A parallel line of research has focused on model compression and efficiency optimization to facilitate the deployment of deep learning models on resource-constrained hardware, a common scenario in agricultural settings.

Lightweight Network Design: The core strategy involves architectural innovations that fundamentally reduce computational complexity. The MobileNet family of architectures (Hoang, 2018; KC et al., 2019; Yang et al., 2019; Mishra et al., 2021; Liu et al., 2022; Liu et al., 2022; Liu et al., 2022; MixCIM: A hybrid-cell-based computing-in-memory macro with less-data-movement and activation-memory-reuse for depthwise separable neural networks, 2024) is a prime example, extensively using depthwise separable convolutions to decouple spatial filtering from channel fusion, thereby reducing both parameter count and computational overhead (FLOPs). This makes them particularly suitable for mobile and embedded deployment. Their efficacy has been demonstrated in various plant disease detection applications (Ling et al., 2020; Mishra et al., 2021; Liu et al., 2022; Liu et al., 2022; Chawla et al., 2024). Similarly, ShuffleNet (Anitha et al., 2024) optimizes lightweight performance by introducing a channel shuffle operation that enhances information flow between feature channels while maintaining low computational cost.

Model Compression Techniques: Beyond inherently lightweight architectures, strategies such as knowledge distillation (Xie et al., 2020; Yun et al., 2024; Cao et al., 2025; He et al., 2025) and model pruning (Li et al., 2024; Huang et al., 2025; Zhang et al., 2025) have been used to compress existing models. Knowledge distillation transfers knowledge from a large, accurate teacher model to a smaller, efficient student model, often with minimal performance loss (Shi et al., 2024). Pruning methods systematically remove unimportant weights or neurons from a trained model, effectively reducing its size and accelerating inference. These strategies are crucial for adapting powerful models to run efficiently on edge devices such as smartphones and embedded systems widely used in precision agriculture.

Data Enhancement and Transfer Learning: To overcome challenges related to limited or imbalanced datasets, data augmentation techniques (Rajasekaran et al., 2020; Chen et al., 2023; Yu et al., 2023; Bouacida et al., 2024; Chen et al., 2024; Prasath et al., 2024) are widely employed. Transformations such as rotation, flipping, cropping, and color adjustments increase training data diversity, thereby improving model generalization to unseen conditions. Transfer learning (Rajasekaran et al., 2020; Mishra et al., 2021; Bonkra et al., 2023; Velagala et al., 2024; Yang et al., 2024; Venkatramulu et al., 2025), which involves fine-tuning models pre-trained on large-scale datasets such as ImageNet, is another common approach. This technique allows for rapid adaptation to new tasks, such as novel plant disease detection, while reducing dependence on extensive labeled datasets and shortening training times.

The application of YOLO models also extends beyond close-range phenotyping to broader agricultural remote sensing tasks. For example, YOLOv8 has been successfully adapted for land-cover classification, fundamental to identifying and mapping agricultural fields from aerial and satellite imagery (Zhang et al., 2025). These large-scale applications demonstrate the architecture’s robustness in processing complex environmental scenes and its versatility across spatial scales. This proven capability in macro-scale agricultural monitoring further supports adapting and optimizing the YOLO framework for specialized, fine-grained tasks such as litchi variety detection.

Despite advances in agricultural AI, lightweight model design, and YOLO evolution, a critical gap persists in developing a specialized model for the fine-grained visual classification of litchi varieties. Existing state-of-the-art models are typically designed for general-purpose detection and are often computationally prohibitive for real-time mobile deployment. More importantly, they lack the architectural inductive biases necessary to capture and analyze the subtle microtextural patterns, color gradients, and morphological features essential for distinguishing between highly similar varieties such as “Icy-Flesh Litchi” and “Osmanthus-Fragr Litchi.” Furthermore, the absence of a large-scale, standardized, publicly available benchmark dataset for litchi variety identification has hindered comparative research and progress in this field.

Motivated by the urgent industrial demand for an efficient, accurate, and practical post-harvest identification system, this study aims to bridge this gap. Our work pursues the dual objectives of achieving high-precision discrimination and real-time performance suitable for edge devices. To this end, we construct a comprehensive litchi image dataset as a benchmark and propose the YOLO-LitchiVar model. Based on the YOLOv12 architecture, our model introduces a synergistic triple-module optimization strategy: (1) the DSC3k2 module for foundational lightweighting, (2) the C2PSA module for enhanced multi-scale feature aggregation focused on shallow textures, and (3) the ECA attention mechanism for channel-wise feature recalibration to suppress noise and amplify discriminative features. This integrated approach is designed to overcome the identified limitations and establish a new performance standard in automated litchi variety identification.

## 3 Materials and methods

### 3.1 Dataset construction and processing

#### 3.1.1 Image acquisition

The variety identification methodology presented in this study demonstrates significant originality and scientific value. Litchi samples were sourced from the core production area in Maoming, Guangdong, China, during the ripening seasons (May to July) of 2024 and 2025. High-precision imaging equipment was used to capture images of multiple varieties, including “Osmanthus-Fragr Litchi” and “Concubine-Smile Litchi,” from various angles. All image acquisition was conducted in a controlled laboratory setting to simulate typical post-harvest inspection environments.

To ensure sample diversity and model robustness, litchi samples were collected at their optimal commercial maturity stages during the respective harvesting seasons (May to July). While maturity levels were standardized within each variety’s harvesting window, the dataset also encompasses natural variations in fruit size, color saturation, and surface characteristics that occur even at commercial maturity. This approach enhances the model’s generalization capability to real-world post-harvest scenarios while maintaining consistency in sample quality.

**Figure 1:**
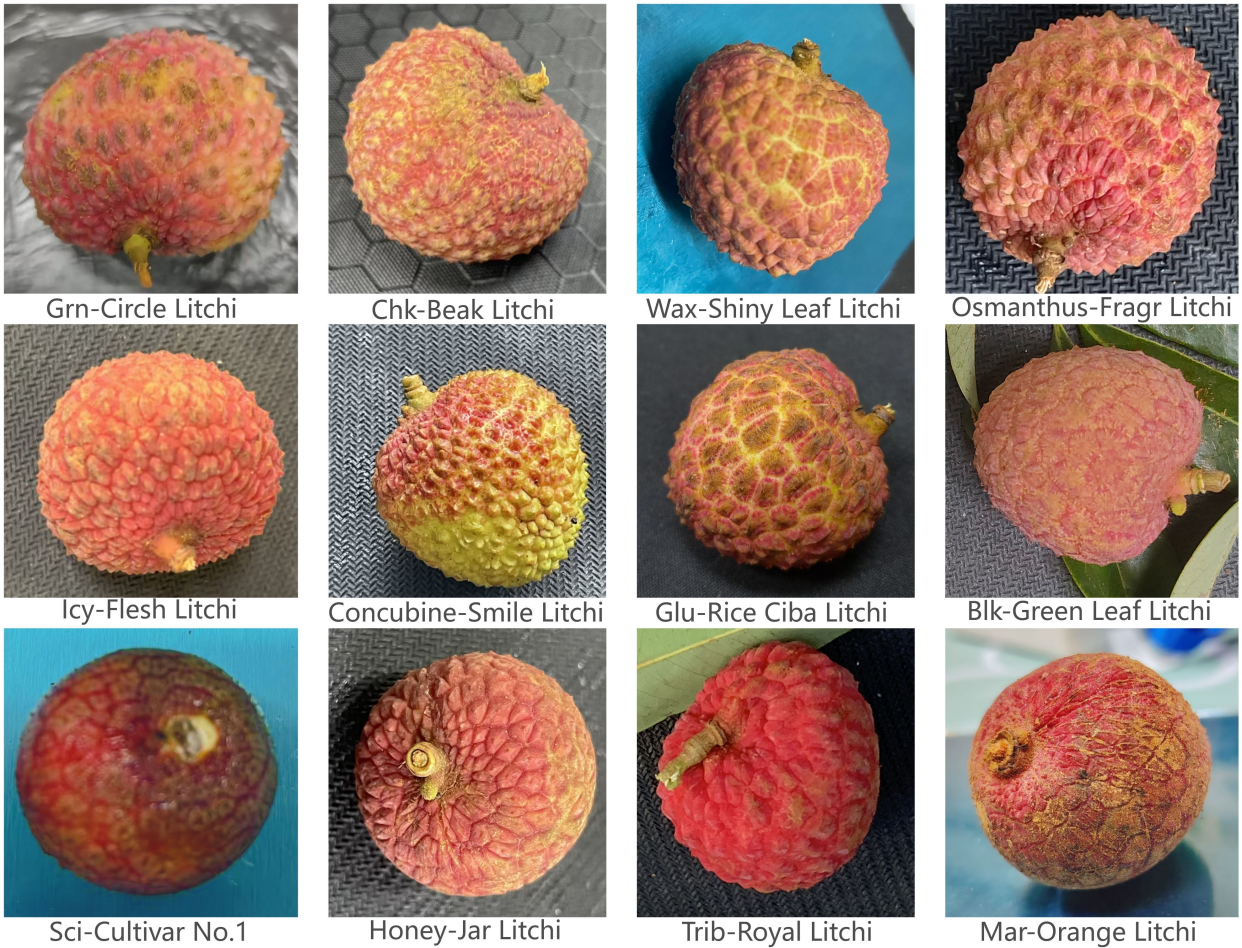
Twelve Litchi varieties included in this study.

Although the background was not perfectly uniform, this setup accurately represents typical post-harvest handling environments and preserves the authentic appearance of different litchi varieties. A total of 11,998 images were collected, providing a rich and reliable primary dataset for this study. **Figure 1** shows representative samples of the 12 varieties included.

To simulate the device diversity encountered in practical applications, this study used four mainstream smartphones as imaging devices and set their key camera parameters as follows: using a prime lens (equivalent focal length ≈ 26 mm), ISO set to auto (automatically determined by the device according to the ambient light), white balance set to automatic white balance (AWB), and the exposure mode set to automatic exposure mode (P/Auto); all the captured images were set to automatic exposure mode (P/E). Auto); all captured images are saved in high-quality JPEG format at the default output resolution of the device (usually the high signal-to-noise resolution in Binning mode). The acquisition device specifications and default settings are shown in **Table 1**. Note that the sensor size, pixel size, and aperture directly affect the signal-to-noise ratio (especially in low light), depth of field, and ability to capture detail.

**Table 1:** Specifications and default settings for image capture devices.

Parameter/model	iPhone 12	Honor 50	Honor X50	Realme GT Neo (Speed edition)
Primary sensor model	Apple custom	Samsung HM2 (108MP)	Samsung HM6 (108MP)	Sony IMX682 (64MP)
Effective resolution	12 MP (Default)	12 MP (9-in-1 binning) 108 MP (Native)	12 MP (9-in-1 binning) 108 MP (Native)	16 MP (4-in-1 binning) 64 MP (Native)
Default output resolution	4032 × 3024 px	4000 × 3000 px (binned)	4000 × 3000 px (binned)	4624 × 3468 px (binned)
Native high-res mode	Not supported	12032 × 9024 px	12000 × 9000 px	9280 × 6944 px
Sensor size (inch)	1/2.55”	1/1.52”	1/1.67”	1/1.73”
Pixel size (μm)	1.4 (Native)	2.1 (binned)	1.92 (binned)	1.6 (binned)
Aperture (f/)	f/1.6	f/1.9	f/1.75	f/1.8
Key features	Smart HDR 3, deep fusion	Multi-frame AI enhancement	Multi-frame noise reduction	AI scene detection

#### 3.1.2 Data labeling and storage

**Figure 2:**
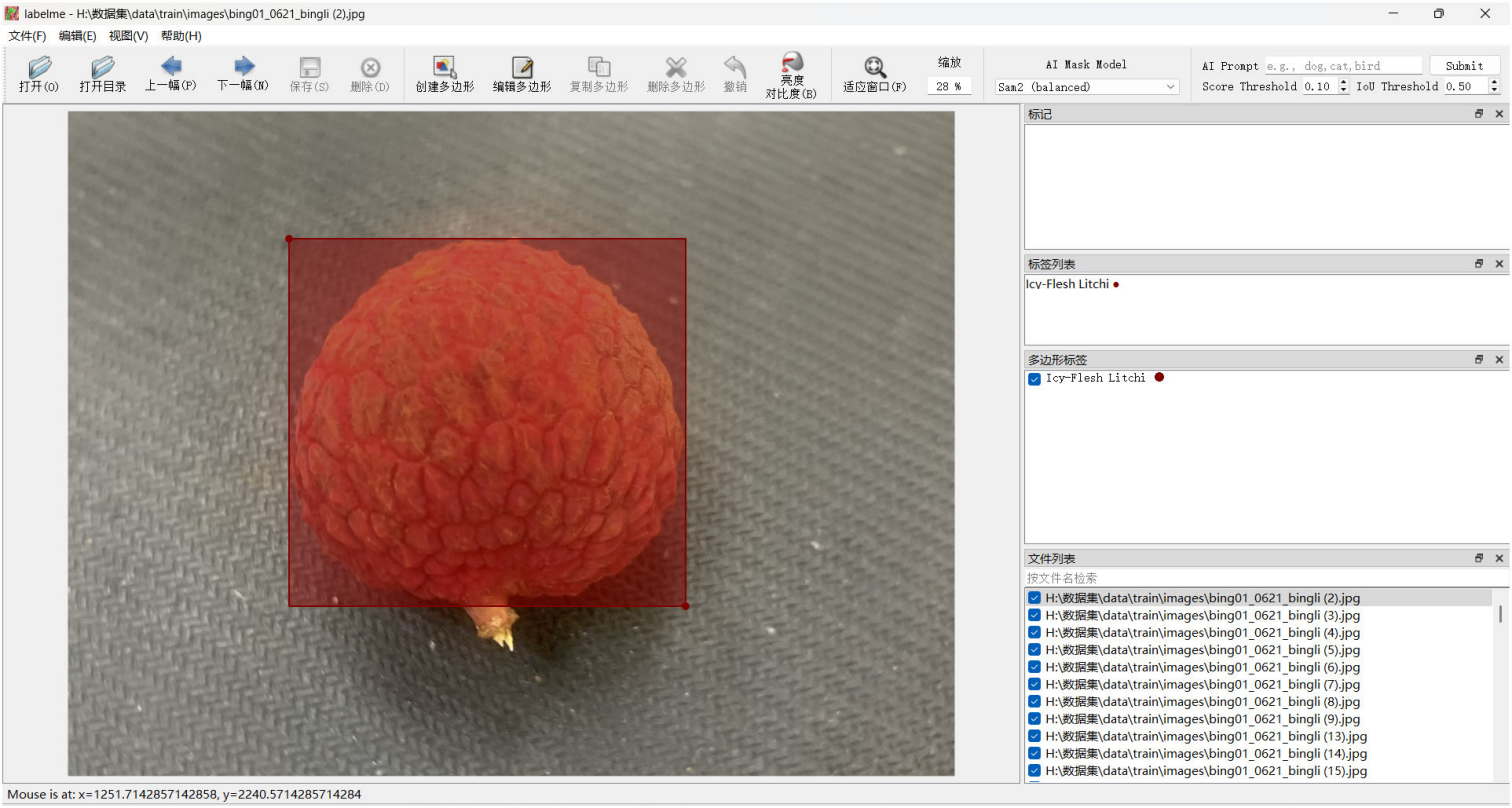
Labeling interface.

All images were manually annotated using the LabelImg tool, generating annotation files in JSON format. The annotation process followed a standardized protocol, and the precise coordinates of each detection frame and its corresponding variety category were recorded in detail (**Figure 2**). The specific formats of the JSON annotation file and the converted TXT label file are demonstrated in **Box 1** and **Box 2**, respectively.

To ensure annotation consistency across the dataset, standardized specifications and procedures were developed and strictly implemented. To comply with the requirements of the subsequent model training framework, automated scripts were created to efficiently and accurately batch-convert the JSON-format annotations into standard TXT-format label files. All converted label files follow uniform, traceable naming conventions and are stored separately within a structured, dedicated label directory.

This rigorous annotation and standardized conversion process ensure data accuracy, format consistency, and ease of use from the outset, providing a solid and reliable foundation for training and evaluating the high-performance litchi variety detection model.

①Example of main content of JSON file

{

“version”: “5.8.1”, “flags”: {}

“flags”: {},

“shapes”: [

{

“label”: “Icy-Flesh Litchi”, “points”: [{

“points”: [

[

1187.4285714285716,.

629.8571428571429

],

[

2912.428571428571, [

2404.8571428571427

]

],

“group_id”: null,

“description”: ““, “shape_type”: “rectangle”, “shape_type”.

“shape_type”: “rectangle”,

“flags”: {},

“mask”: null

}

……

}

② Example of the main content of a txt file

4 0.508415 0.501772 0.427827 0.586971

#### 3.1.3 Dataset composition and characteristics

The constructed dataset is comprehensive and designed to support the development of a robust litchi variety detection model. It comprises a total of 11,998 high-quality images covering 12 common commercial litchi varieties, as illustrated in **Figure 1**. The included varieties are Osmanthus-Fragr Litchi, Glu-Rice Ciba Litchi, Trib-Royal Litchi, Chk-Beak Litchi, Icy-Flesh Litchi, Wax-Shiny Leaf Litchi, Grn-Circle Litchi, Mar-Orange Litchi, Honey-Jar Litchi, Concubine-Smile Litchi, Blk-Green Leaf Litchi, and Sci-Cultivar No. 1.

Images were captured under controlled laboratory conditions simulating a post-harvest environment, using natural indoor lighting. Although the background was not perfectly uniform, this setting enhances the dataset’s realism and applicability to practical scenarios. To further improve the generalizability of the trained model and simulate device diversity encountered in real-world applications, images were acquired using four mainstream smartphone models (iPhone 12, Honor 50, Honor X50, and realme GT Neo Speed Edition) with their default automatic camera settings. The detailed specifications of these imaging devices are summarized in **Table 1**. The dataset was meticulously partitioned into a training set (8,199 images, ~70%), a validation set (2,331 images, ~20%), and a test set (1,468 images, ~10%) to facilitate a rigorous model development and evaluation workflow.

### 3.2 YOLO-LitchiVar model architecture

#### 3.2.1 YOLOv12 baseline structure

**Figure 3:**
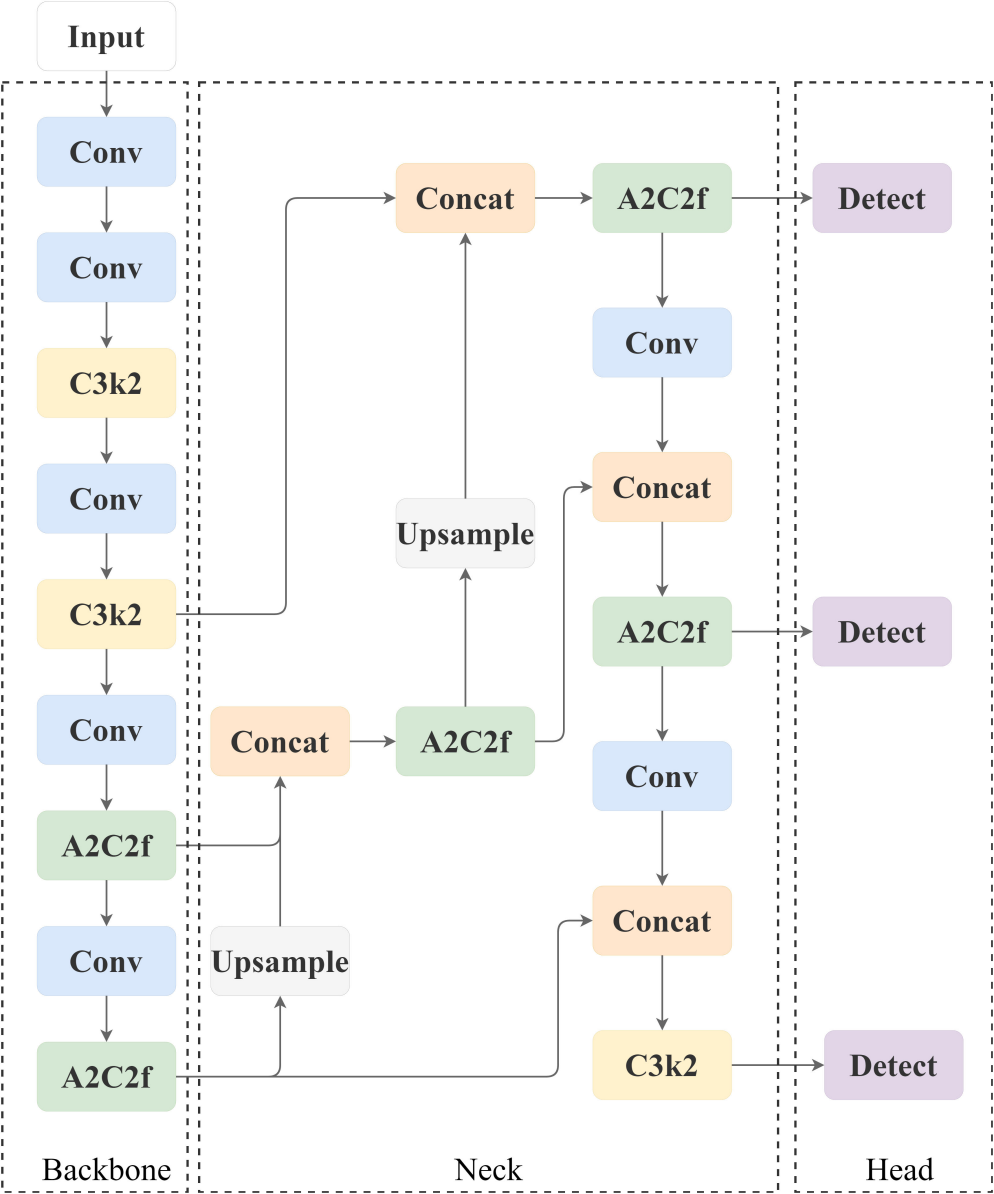
Network structure of YOLOv12.

The core architecture of YOLOv12 follows the classic design principles of the YOLO series. **Figure 3** shows the three-part composition of YOLOv12, which includes the backbone network, neck network, and head network.

##### 3.2.1.1 Backbone network (backbone)

The backbone network is responsible for extracting multi-scale and multi-level features from the input image. Through a series of well-designed convolutional layers, down-sampling layers, and innovative modules (e.g., C3k2, A2C2f), it gradually reduces the spatial resolution of the feature map while increasing its channel depth to capture rich information—from low-level texture to high-level semantics. The backbone outputs multiple feature maps at different scales, representing features under various receptive fields.

The convolutional layers within these modules follow a standardized configuration: spatial feature extraction uses 3 × 3 kernels, while channel-dimension manipulation uses 1 × 1 kernels. A stride of 1 is applied to convolutions that preserve spatial dimensions, and a stride of 2 is used for down-sampling layers. The SiLU (Sigmoid Linear Unit) activation function is applied uniformly across all convolutional layers for its non-saturating and smooth-gradient properties, which improve training dynamics and feature representation.

###### 3.2.1.1.1 Core module C3k2

The core structure of the C3k2 module consists of multiple “Convolutional Layer (Conv) + Batch Normalization Layer (BN) + SiLU Activation Function” stacked together, and introduces a residual connection that adds the module input directly to the processed feature maps. This design enables multi-scale feature extraction, significantly enhancing feature expression. The residual structure alleviates gradient-vanishing issues in deep networks and stabilizes feature propagation. In the backbone, stacking these modules progressively expands the receptive field, capturing richer global contextual information. Ultimately, the optimized features output from this module are further utilized in the subsequent feature fusion stage, which significantly improves the discriminative properties of the features and provides a crucial high-quality feature base for high-precision target recognition across the detection network.

###### 3.2.1.1.2 Core module A2C2f

The A2C2f (Area-Attention Enhanced Cross-Feature) module is an improved feature-extraction block introduced in YOLOv12. It combines area attention and residual connection mechanisms to improve feature-extraction efficiency and accuracy. The A2C2f module consists of the following components:

①cv1 and cv2: two layers of 1×1 convolution, which are used for the downscaling of input features and upscaling of output features, respectively.

②ABlock module: The core of A2C2f, containing area-attention and MLP (multi-layer perceptron) layers for rapid feature extraction and attention enhancement.

③Residual Connection: optional residual connection for stabilizing training and enhancing feature representation.

##### 3.2.1.2 Neck network (neck)

Neck Network: assumes the key role of feature fusion and enhancement. It receives the multi-scale feature maps from the backbone and uses up-sampling, concatenation, and specific fusion modules (e.g., A2C2f) to aggregate features across scales. This multi-scale fusion strategy combines high-resolution feature maps (rich in spatial detail) with low-resolution maps (strong in semantic information), thereby improving the model’s ability to detect objects of different sizes, particularly small targets.

##### 3.2.1.3 Head network (head)

Head Network: The final target localization and classification prediction is based on the fused features. It typically consists of several convolutional layers responsible for generating bounding-box coordinates, the Confidence of the target, and the Class Probabilities.YOLOv12’s head is designed to be efficient and able to quickly output dense prediction results.

##### 3.2.1.4 Key architecture

The core advantage of YOLOv12 over its predecessor lies in key architectural innovations designed to balance accuracy and speed in real-time target detection and to optimize the training process:

(1)A2 module (Area attention module): Traditional global self-attention mechanisms have high computational complexity (O (N²)), limiting real-time application. YOLOv12’s A2 module divides the feature map into equal-sized, non-overlapping horizontal or vertical blocks and computes self-attention within each block. Segmentation is achieved via a simple reshape operation, avoiding the overhead of complex windowing mechanisms. This approach significantly reduces computational cost while retaining a large effective receptive field, surpassing traditional local-attention methods and improving processing efficiency without sacrificing accuracy.

(2) R-ELAN (Residual-based efficient layer aggregation network): YOLOv12 employs advanced feature-aggregation modules within its backbone, including ELAN (Efficient Layer Aggregation Network) and its enhanced variant R-ELAN (Tian et al., 2024). These modules strengthen gradient flow and enrich feature representation through multi-branch concatenation structures. Specifically, R-ELAN introduces a block-level residual connection strategy with scaling techniques and a redesigned aggregation pathway to overcome optimization challenges and ensure stable convergence in deep networks. For detailed architectural information, readers are referred to the original technical report (Tian et al., 2024). These established components form a robust foundation that allows the proposed novel modules (DSC3k2, C2PSA, ECA) to focus on lightweight optimization and fine-grained discrimination of litchi varieties.

#### 3.2.2 YOLO-LitchiVar model architecture

To systematically address the identified limitations pertaining to model complexity, shallow feature underutilization, and interclass discriminability, this study proposes a structurally enhanced variant—YOLO-LitchiVar—built upon the YOLOv12 architecture. The model design integrates three novel, synergistic modules: a Depthwise Separable Convolutional (DSC3k2) module for foundational lightweighting, a Cross-level to Progressive Scale Aggregation (C2PSA) module for hierarchical feature enrichment, and an Efficient Channel Attention (ECA) module for adaptive feature recalibration. This multi-faceted refinement is explicitly engineered to achieve a superior balance between computational efficiency and discriminative accuracy for fine-grained litchi variety detection.

The YOLO-LitchiVar model builds upon the YOLOv12-nano architecture with targeted modifications to balance performance and efficiency. The backbone network incorporates DSC3k2 modules at strategic locations to reduce computational complexity while maintaining feature-extraction capability. Specifically, standard convolutional layers in the early stages are replaced with DSC3k2 modules to achieve preliminary parameter compression. The C2PSA module is integrated at the P5/32 output level of the backbone to facilitate cross-scale feature aggregation before the detection head. The ECA attention mechanism is applied in both the backbone (after C2PSA) and the detection head (P5/32 level) to enable channel-wise feature recalibration at critical network stages. This architectural design ensures that lightweight optimization, feature enhancement, and attention mechanisms function synergistically throughout the network hierarchy.

**Figure 4:**
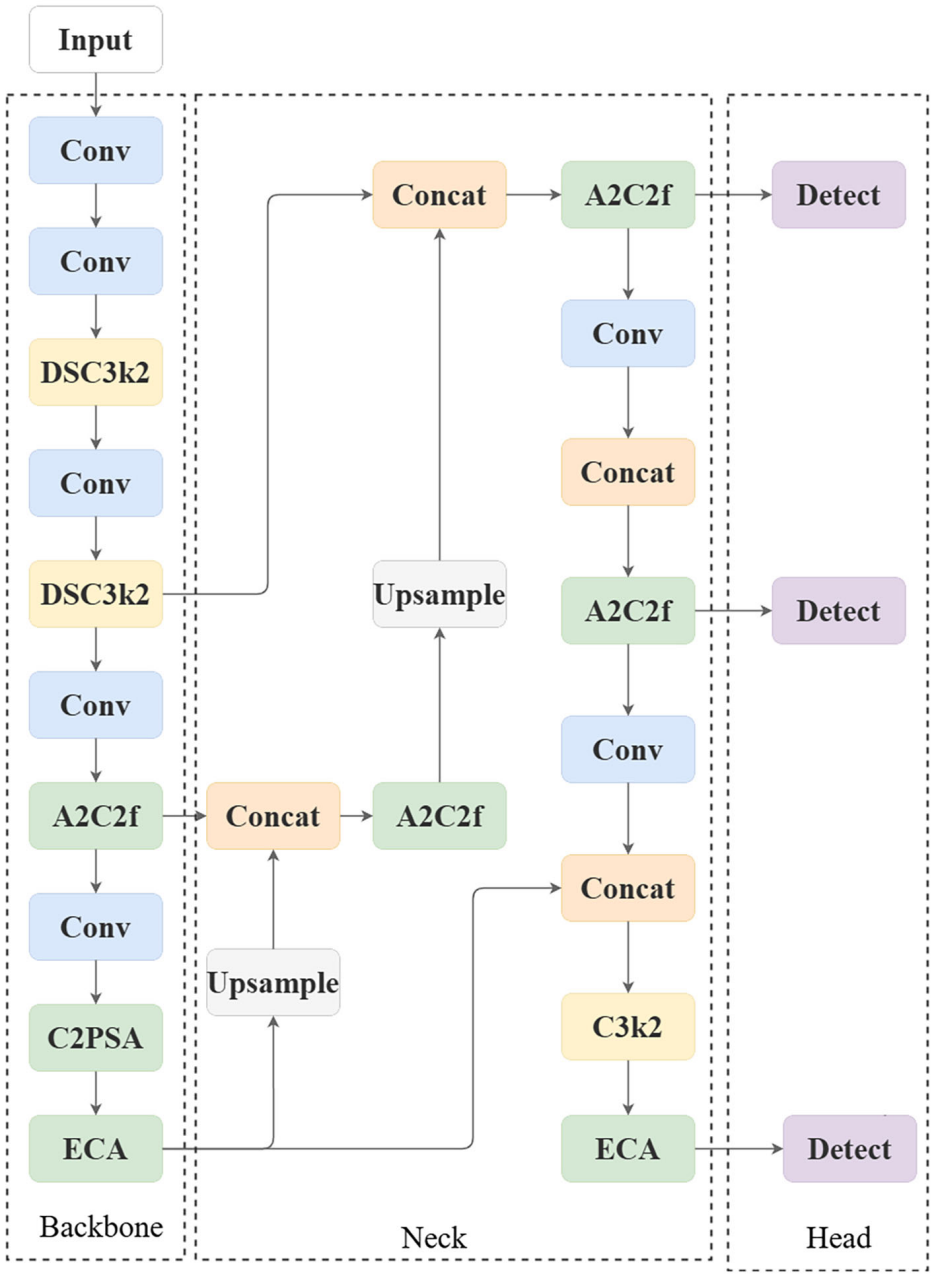
Three-module synergistic architecture.

The architectural innovation of YOLO-LitchiVar is characterized by the principled integration and cascaded operation of the DSC3k2, C2PSA, and ECA modules. Each module mitigates a specific performance bottleneck: DSC3k2 primarily targets parameter and FLOPs reduction; C2PSA addresses the semantic gap and spatial-detail loss across network hierarchies; and ECA enhances feature selectivity against noise and interclass similarity. Their sequential application forms a coherent pipeline that progressively refines feature representations, enabling significant gains in both efficiency and accuracy. The overall structure of the YOLO-LitchiVar model is shown in **Figure 4**.

##### 3.2.2.1 Backbone optimization

①Lightweight backbone replacement: Standard convolutions in specific layers of the backbone network are replaced with DSC3k2 modules to realize parameter compression and computation reduction.

②Feature fusion enhancement: C2PSA modules are strategically inserted at the backbone output or along multi-layer feature-transfer paths to strengthen cross-layer aggregation—particularly shallow P3—enhancing the characterization of microtextures such as Icy-Flesh Litchi LBP features.

##### 3.2.2.2 Neck and head optimization

**Figure 5:**
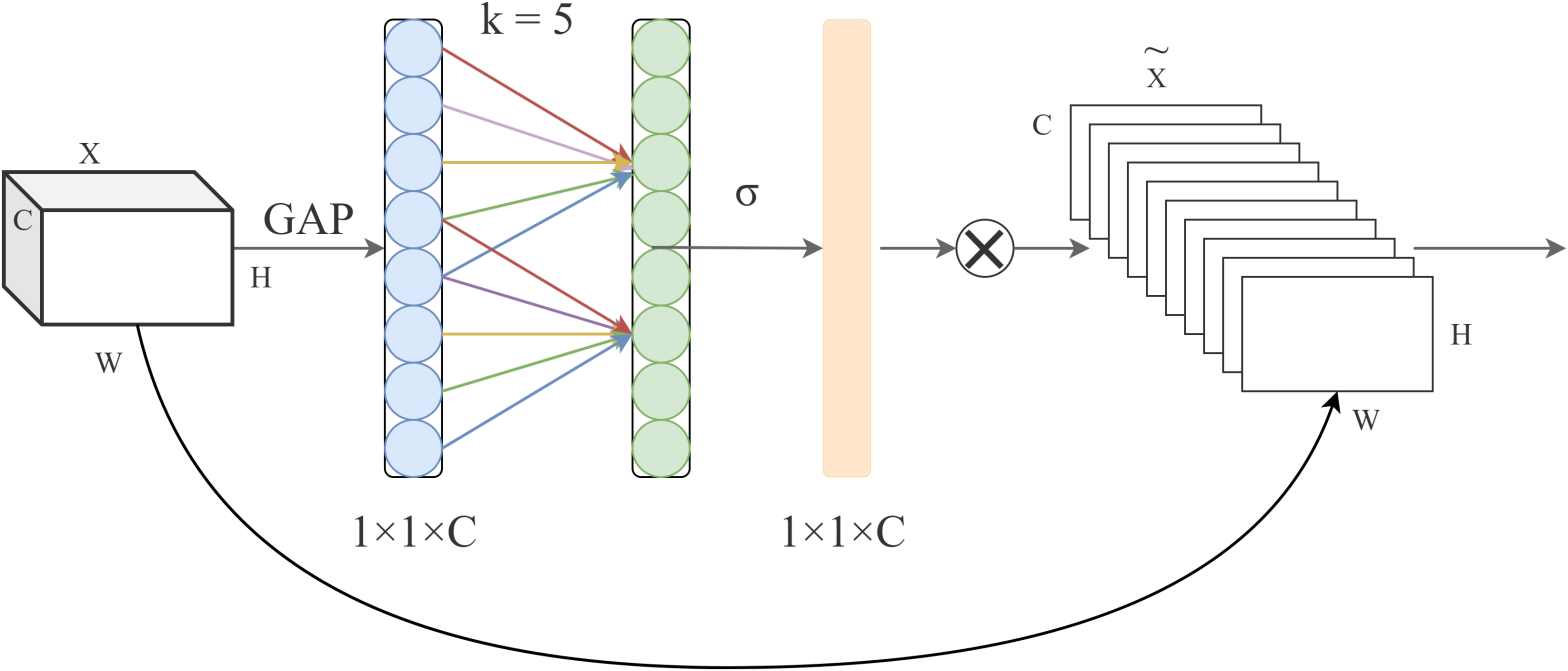
Structure diagram of ECA network.

①Channel attention integration: An ECA module is incorporated within the head network following the feature-fusion stages of the neck. This placement (as corrected in **Figure 5**) allows the attention mechanism to process refined, multi-scale features immediately before the final detection stage, ensuring optimal channel-wise recalibration for classification and localization. This strategic placement allows the module to process the multi-scale, semantically rich, and spatially refined feature maps produced by the Neck network before they are used for the final prediction tasks (localization and classification). The module operates on these fused features, analyzing the collective contribution of each channel across all aggregated scales. By performing channel-wise recalibration at this stage, the ECA mechanism effectively prioritizes channels that contain discriminative information relevant to the specific litchi variety present in the receptive field, while attenuating channels that respond to confounding factors like varying lighting conditions or shared background elements. This adaptive, input-dependent conditioning guides the detection head toward more accurate and robust predictions.

②Module synergistic connection: Features extracted and initially fused by the backbone are further integrated by the neck and then input into the ECA module for channel optimization. The optimized features are finally passed to the detection head for target localization and classification. In this cascade, the DSC3k2 module establishes the lightweight foundation and preserves key features; C2PSA enhances detailed characterization—especially for Icy-Flesh Litchi microtextures; and ECA refines the resulting features by focusing on discriminative information.

#### 3.2.3 Three core modules

##### 3.2.3.1 DSC3k2 module: depth-separable convolutional lightweighting

**Figure 6:**
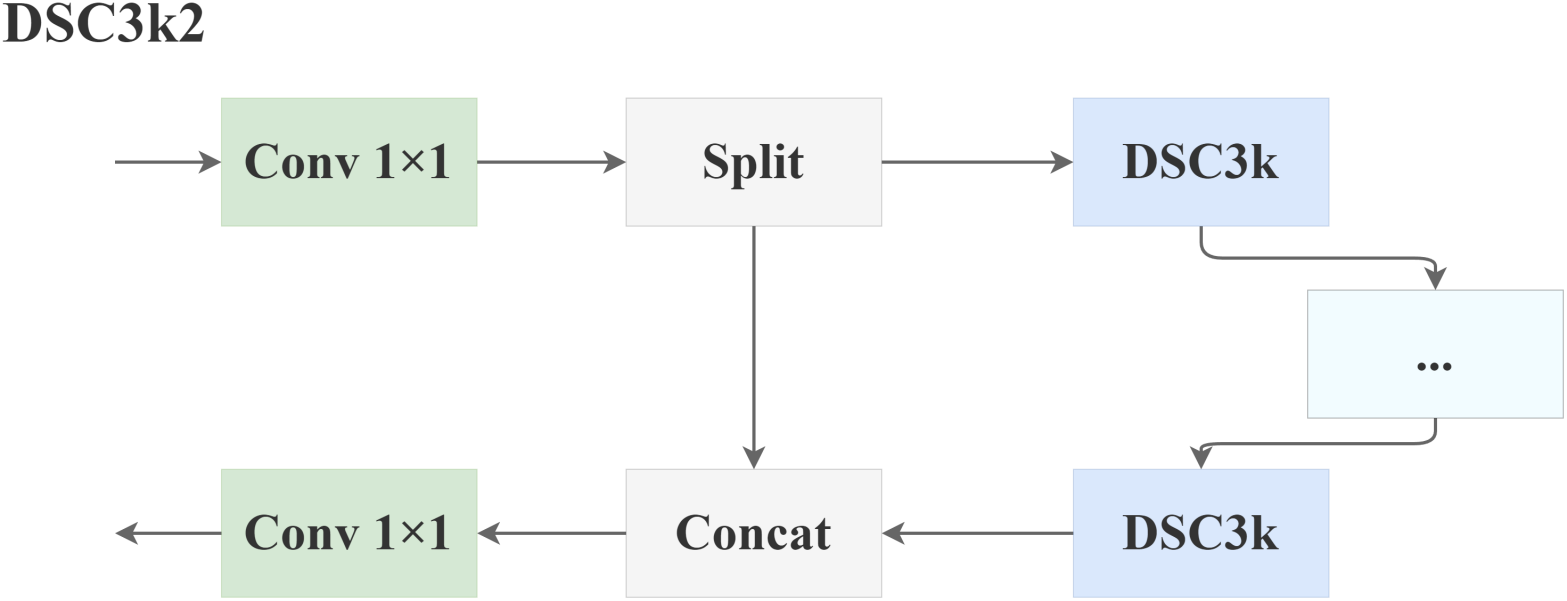
Structure of the DSC3k2 network.

The DSC3k2 module is architected to achieve substantial model compression, serving as the primary mechanism for reducing computational overhead and parameter count. Its detailed structure, depicted in **Figure 6**, extends the basic principle of depthwise-separable convolution by incorporating a parallel-processing design. The input features are first split into multiple branches. Each branch independently processes the features through a dedicated depthwise-separable convolutional (DSC) sub-module, enabling a more diverse feature transformation. The outputs from these parallel branches are subsequently concatenated to synthesize a comprehensive feature representation. This Split *→* Parallel DSC *→* Concat design enhances representational capacity beyond the basic DSC operation while maintaining its lightweight advantage through spatial and channel decoupling (Lei et al., 2024).

Structure definition and lightweighting principle:

###### 3.2.3.1.1 Comparison of the number of parameters

Standard convolution—parameter number (Equation 1):

(1) $$\begin{array}{c} {\text{Params}}_{\text{std}}={\text{C}}_{\text{in}}\times {\text{C}}_{\text{out}}\times {\text{K}}^{2}+{\text{C}}_{\text{out}}(\text{K}=3) \end{array}$$

DSC3k2 parametric quantity (Equation 2):

(2) $$\begin{array}{c} {\text{Params}}_{\text{DSC}3\text{k}2}={\text{C}}_{\text{in}}\times {\text{K}}^{2}+{\text{C}}_{\text{in}}\times {\text{C}}_{\text{out}}+{\text{C}}_{\text{out}} \end{array}$$

${\text{C}}_{\text{in}}$ denotes the number of input channels, ${\text{C}}_{\text{out}}$ is the number of output channels, and K is the size of the convolution kernel. In deep convolution, each input channel corresponds to one convolution kernel for spatial filtering, so the number of parameters is determined by the number of input channels and the size of convolution kernel. The point-by-point convolution is a 1×1 convolution, which is used to fuse the channel information of the deep convolution output, and its number of parameters is determined by the number of input and output channels.

###### 3.2.3.1.2 Compression rate calculation

Parameter amount reduction rate (Equation 3):

(3) $$\begin{array}{c} \text{η}=1-\frac{{\text{C}}_{\text{in}}({\text{K}}^{2}+{\text{C}}_{\text{out}})}{{\text{C}}_{\text{in}}{\text{C}}_{\text{out}}{\text{K}}^{2}}=1-\frac{1}{{\text{C}}_{\text{out}}} \end{array}$$

This formula is used to measure the parameter compression ratio of the depth separable convolution (DSC) relative to the standard convolution. The formula shows that when the number of output channels ${\text{C}}_{\text{out}}$ is large, the compression ratio will be close to 1, i.e., it can significantly reduce the model parameters and decrease the model complexity. By decoupling spatial filtering and channel fusion, this structure significantly reduces the model complexity while maintaining the effective feature extraction capability.

##### 3.2.3.2 C2PSA module: cross-layer feature aggregation enhancement

The design motivation for the C2PSA module stems from the critical need to address information loss in fine-grained litchi variety discrimination. Shallow feature maps (P3) preserve high-resolution spatial details, including the micro-concave textures characteristic of *Icy*-Flesh *Litchi*, but lack semantic richness. Deeper feature maps (P5) contain strong semantic information but suffer from coarse spatial resolution. The P4 layer provides an intermediate representation. Integrating the P3, P4, and P5 layers enables comprehensive feature representation spanning detailed textures to high-level semantics.

The “dimensionality reduction first, then alignment, finally concatenation and fusion” strategy was adopted for several reasons. First, the use of 1×1 convolution for channel reduction before alignment minimizes computational overhead during up-sampling and down-sampling operations. Second, this sequential processing ensures that feature maps at different scales maintain their distinctive characteristics while being prepared for effective fusion. Finally, the 3×3 convolution after concatenation facilitates smooth integration of multi-scale features.

Compared with traditional feature pyramid architectures, C2PSA offers distinct advantages:

1. Unlike FPN, which primarily focuses on top-down semantic enhancement, C2PSA implements bidirectional cross-scale interaction.
2. Compared with PANet’s complex path augmentation, C2PSA maintains efficiency while achieving effective feature alignment.
3. Relative to BiFPN’s weighted feature fusion, C2PSA employs a simpler yet effective concatenation-based approach that is sufficient for capturing subtle inter-variety differences in litchi recognition.

This design specifically addresses the challenge of preserving microtextural information throughout the network hierarchy, which is crucial for distinguishing visually similar litchi varieties.

The C2PSA module is designed to mitigate the inherent information loss that occurs as feature maps undergo progressive down-sampling within the network backbone. Shallow feature maps (e.g., P3) retain high spatial resolution and rich low-level textural details—such as the micro-concave pericarp patterns of *Icy*-Flesh *Litchi*—but possess weaker semantic information. Conversely, deeper feature maps (e.g., P4, P5) encode stronger semantic concepts but have lower spatial resolution.

The C2PSA module explicitly addresses this semantic–spatial discrepancy through a structured multi-scale feature alignment and fusion strategy. It aggregates feature maps from different hierarchical levels (typically P3, P4, P5), aligning them to a common spatial scale (usually that of the highest-resolution map, P3) using a combination of up-sampling and down-sampling operations. After channel reduction via 1×1 convolutions to ensure dimensional compatibility, the aligned features are concatenated. A final 3×3 convolutional layer is then applied to seamlessly fuse the concatenated multi-scale features, synthesizing a new feature representation that simultaneously incorporates fine-grained spatial detail from shallow layers and robust semantic context from deeper layers.

Feature fusion mechanism (Equation 4):

(4) P3'=Conv1x1(P3)P4'=U2x(Conv1x1(P4))P5'=D2x(Conv1x1(P5))Pout=Conv3x3(Concat [P3',P4',P5'])

where U denotes upsampling and D denotes downsampling to achieve cross-layer interaction between shallow texture and deep semantics.

- 1x1 convolution: applied to feature maps (P3, P4, P5) from different layers of the backbone network Uniform number of channels.
- 2x upsampling of P4 (U_2x_) and 2x downsampling of P5 (D_2x_): performed to align feature map resolutions with P3.
  - The processed P3′, P4′, and P5′ are concatenated (Concat).
  - Finally, the concatenated features are fused through a **3×3** convolution (Conv_3_x_3_) to output the enhanced feature map P_out. This mechanism effectively integrates shallow, high-resolution texture information with deep, semantically rich information, facilitating the capture of *Icy-Flesh Litchi* micro-concave LBP features and improving overall variety discrimination.

**Figure 7:**
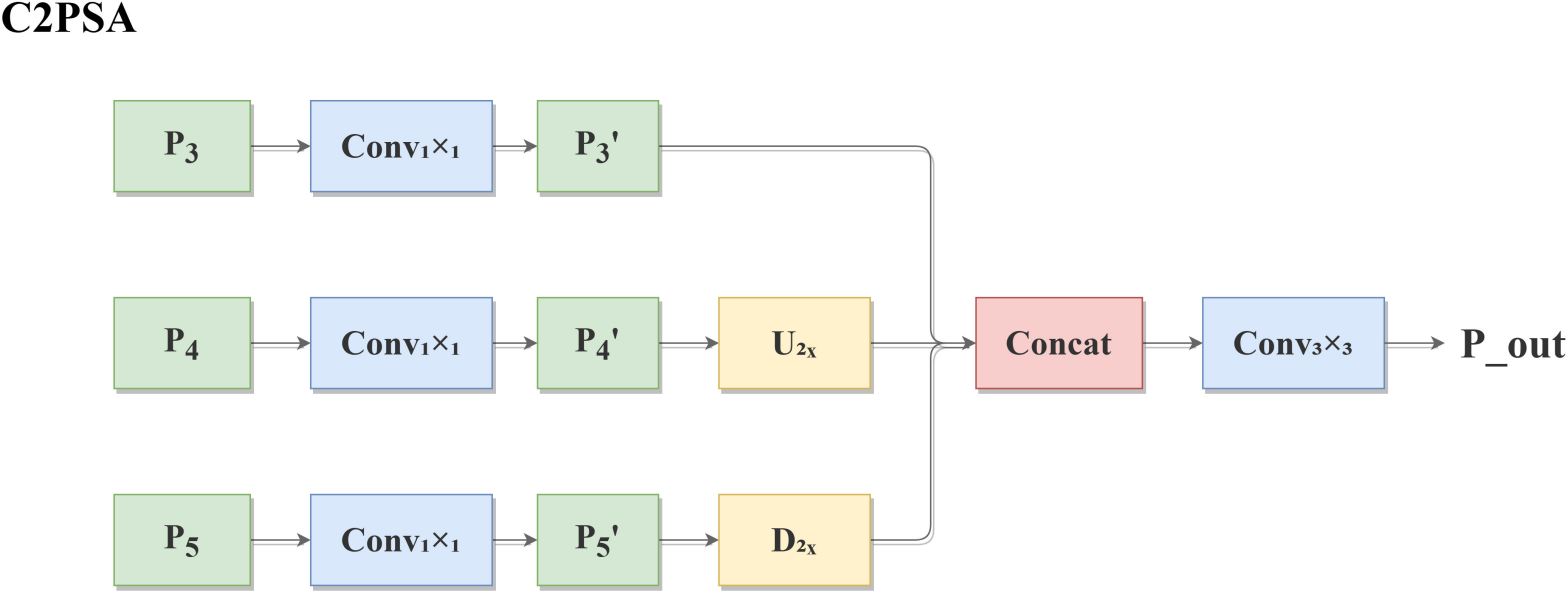
Structure of the C2PSA network.

The structure of the C2PSA module is shown in **Figure 7**.

##### 3.2.3.3 ECA module: efficient channel attention

The ECA module introduces a lightweight, channel-wise attention mechanism to dynamically recalibrate feature responses, thereby enhancing the network’s representational power. Its operation is based on the principle that not all feature channels contribute equally to the discriminative task for a given input.

The module first generates a channel-wise descriptor vector by applying Global Average Pooling (GAP) to squeeze global spatial information from each channel. Crucially, instead of using computationally expensive fully connected layers to capture channel dependencies, ECA employs a fast 1D convolution with an adaptively determined kernel size. This kernel size is a nonlinear function of the channel dimension, ensuring that the coverage of cross-channel interaction is appropriate for the network’s capacity.

The output of this 1D convolution is passed through a Sigmoid activation function to produce a vector of soft attention weights (each between 0 and 1), corresponding to the relative importance of each feature channel. The final output is obtained by multiplying the original input feature map by these attention weights, effectively amplifying features that are salient for variety discrimination (e.g., specific texture patterns) while suppressing less informative or noisy features (e.g., background variations, illumination artifacts, and features common to confusing varieties), such as Icy-Flesh Litchi microtexture channel and Osmanthus-Fragr Litchi ridge peak channel, and its structure is shown in **Figure 5** (Wang et al., 2019).

###### 3.2.3.3.1 Global average pooling

The input feature map $\text{χ}\in {\text{R}}^{1\times 1\times \text{C}}$, compressed along the spatial dimension, generates channel-level statistics:

(5) $$\begin{array}{c} \text{GAP}({\text{χ}}_{\text{c}})=\frac{1}{\text{H}\times \text{W}}\sum_{\text{i}=1}^{\text{H}}\sum_{\text{j}=1}^{\text{W}}{\text{χ}}_{\text{c}}(\text{i},\text{j}) \end{array}$$

This operation aggregates spatial information and outputs the channel descriptor $\text{Z}\in {\text{R}}^{\text{C}\times 1\times 1}$ (labeled in the figure:GAP operation).

###### 3.2.3.3.2 Local Cross-Channel Interaction

Inter-channel dependencies are captured by 1D convolution with adaptive kernel size (labeled 1DConv).

(6) $$\begin{array}{c} s=Conv1D_{k}(z),k=\varphi (C)=|\frac{\log2C}{\gamma }+\frac{b}{\gamma }{|}_{odd} \end{array}$$

Where |·| odd denotes the closest odd number, and the hyperparameters γ= 2,b=1 control the nonlinear mapping of kernel size k to the number of channels C. The kernel size k and the number of channels C are then mapped to the kernel size.

###### 3.2.3.3.3 Sigmoid activation function

Normalize the interacted features to generate the channel attention weights (labeled in the figure: Sigmoid activation).

(7) $$\begin{array}{c} w=\sigma (s),w\in [0,1] \end{array}$$

###### 3.2.3.3.4 Channel recalibration

The original features are recalibrated according to the weights (labeled in the figure: channel weighting operation).

(8) $$\begin{array}{c} {\text{y}}_{\text{c}}={\text{w}}_{\text{c}}\cdot {\text{χ}}_{\text{c}} \end{array}$$

The complete channel attention calculation process can be expressed as follows.

(9) $$\begin{array}{c} \text{w}=\text{σ}(\text{Conv}1{\text{D}}_{\text{k}}(\text{GAP}(\text{χ}))) \end{array}$$

The convolution kernel parameter $\text{W}\in {\text{R}}^{\text{k}\times 1\times 1}$ realizes cross-channel interaction (labeled in the figure: W weight matrix).

### 3.3 Implementation details

#### 3.3.1 Model training configuration

Regarding training configuration and dataset construction, the model was developed using the Python 3.10.18 programming environment and trained on an NVIDIA GeForce RTX 3060 GPU with the CUDA 12.6 computing platform. The original dataset containing 11,998 images was partitioned into a training set (8,199 images, ~70%), a validation set (2,330 images, ~20%), and a test set (1,469 images, ~10%) to construct a comprehensive model training and evaluation framework. The key training parameters are summarized in **Table 2**.

**Table 2:** Training parameter setting.

Parameter	Setting
imgsz	416*416
epochs	200
batch	32
true	true
momentum	0.937
lr0	0.01
Activation Function	SiLU

The model hyperparameters were carefully optimized: the initial learning rate (lr0) was set to 0.01, momentum to 0.937, batch size to 32, and the input image size (imgsz) standardized to 416 × 416 pixels. Training was conducted for 200 epochs. This combination of parameters balances training efficiency with model generalization capability, ensuring the reliability and validity of the training outcomes.

The input image size (imgsz) was uniformly resized to 416 × 416 pixels. All convolutional layers used kernels of size 3 × 3 for spatial feature extraction and 1 × 1 for channel manipulation. Stride values were set to 1 for same-resolution convolution and 2 for downsampling. The SiLU activation function was employed across all convolutional layers due to its non-saturating nature and improved gradient flow.

#### 3.3.2 Implementation platform and training environment

To ensure the reproducibility of our experiments and provide a clear technical context, this subsection details the software and hardware environments used for model development and training.

Hardware **platform**: All experiments were conducted on a high-performance computing server equipped with an NVIDIA GeForce RTX 3060 GPU (12 GB VRAM). The model training and evaluation processes were executed on this dedicated hardware to ensure consistent performance and timing metrics.

Software **environment**: The server operating system was Ubuntu 22.04 LTS. Model development and training were performed in a Python 3.10.18 environment. The deep learning framework used was PyTorch 2.7.1, built with CUDA 12.6 to leverage GPU acceleration. Key Python libraries included torchvision 0.18.1, opencv-python 4.8.1, numpy 1.26.4, and scikit-learn 1.4.2 for data processing and metric calculation.

Data **annotation tool**: All image annotations were meticulously performed using the LabelMe graphical image annotation tool (version 5.8.1). This tool facilitated precise manual drawing of bounding boxes and assignment of variety labels, generating annotation files in JSON format for each image.

Training **monitoring**: The training process was initiated and monitored via a terminal connection (using WindTerm) to the server. Key training metrics, including loss values and performance indicators on the validation set, were tracked through the standard output logs of the training script. These logs were periodically saved for offline analysis to monitor convergence behavior and identify potential issues such as overfitting.

#### 3.3.3 Loss function formulation

The training objective for YOLO-LitchiVar is optimized through a multi-component loss function that balances localization precision and classification accuracy. The composite loss function is defined as the weighted sum of three key components:

(10) $$\begin{array}{c} L_{total}=\lambda_{box}L_{box}+\lambda_{cls}L_{cls}+\lambda_{dfl}L_{dfl} \end{array}$$

where the weighting coefficients are empirically set to $\lambda_{box}=7.5$, $\lambda_{cls}=0.5$, and $\lambda_{dfl}=1.5$ based on optimal performance in our experimental validation.

The individual loss components are mathematically formulated as follows:

(1) Bounding Box Loss ( $L_{box}$): Employs Complete IoU (CIoU) loss to comprehensively measure the overlap and spatial relationship between predicted and ground-truth bounding boxes:

(11) $$\begin{array}{c} L_{box}=1-IoU+\frac{\rho^{2}(b_{pred},b_{gt})}{c^{2}}+\alpha v \end{array}$$

where:

IoU represents the Intersection over Union between predicted and ground-truth boxes

-
ρ(bpred,bgt) denotes the Euclidean distance between the center points of predicted and ground-truth boxes
-
c is the diagonal length of the smallest enclosing box covering both predicted and ground-truth boxes

v measures the consistency of aspect ratios, defined as $v=\frac{4}{\pi^{2}}{\left(\arctan\frac{w^{gt}}{h^{gt}}-\arctan\frac{w}{h}\right)}^{2}$(2) Classification Loss $(L_{cls}$): Utilizes binary cross-entropy loss for multi-class classification tasks:

(12) $$\begin{array}{c} L_{cls}=-\sum_{c=1}^{C}\sum_{i=1}^{N}[y_{c,i}\log({\hat{y}}_{c,i})+(1-y_{c,i})\log(1-{\hat{y}}_{c,i})] \end{array}$$

where:

-
C is the total number of litchi variety classes (12 in our case)
-
N represents the number of samples

$y_{c,i}$ denotes the ground-truth label (0 or 1) for class c and sample i ${\hat{y}}_{c,i}$ indicates the predicted probability for class c and sample i(3) Distribution Focal Loss ( $L_{dfl}$): Implements a distribution-based approach to model bounding box coordinates as probability distributions, enhancing localization precision:

(13) $$\begin{array}{c} L_{dfl}=-\sum_{j}((1-y_{j})\log(1-{\hat{y}}_{j})+y_{j}\log({\hat{y}}_{j})) \end{array}$$

This formulation treats bounding box regression as a classification problem over discrete coordinate bins, where $y_{j}$ represents the target distribution and ${\hat{y}}_{j}$ denotes the predicted distribution probabilities.

The synergistic optimization of these three loss components ensures robust feature learning, with Lbox focusing on precise localization, Lcls enhancing variety discrimination, and $L_{dfl}$ refining bounding box coordinate estimation through distribution learning.

## 4 Experiments and results

### 4.1 Experimental setup and evaluation metrics

#### 4.1.1 Evaluation metrics

Model performance evaluation is a core aspect of the target detection task. In order to objectively quantify the performance of the proposed models, this study adopts Precision (P), Recall (R), mean Average Precision (mAP), and mAP50–95 as the main evaluation metrics (Everingham et al., 2010; Hosang et al., 2016), defined as follows:

(1) Precision (P): reflects the proportion of samples predicted by the model to belong to positive categories that are truly positive, calculated as (Equation 14):

(14) $$\begin{array}{c} \text{P}=\frac{\text{TP}}{\text{TP}+\text{FP}} \end{array}$$

where TP (True Positive) is the number of true positives and FP (False Positive) is the number of false positives.

(2) Recall (R): reflects the proportion of samples that are true positive categories correctly predicted by the model, calculated as (Equation 15):

(15) $$\begin{array}{c} \text{R}=\frac{\text{TP}}{\text{TP}+\text{FN}} \end{array}$$

Mean Average Precision (mAP): The mean average precision (AP) of a single category, i.e., the area under the precision-recall curve (PR curve), is first calculated as (Equation 16):

(16) $$\begin{array}{c} \text{AP}=\int_{0}^{1}\text{P}(\text{R})\text{dR} \end{array}$$

Subsequently, the AP of all categories is averaged to obtain mAP (Equation 17):

(17) $$\begin{array}{c} \text{mAP}=\frac{\sum_{i=1}^{N}AP_{i}}{\text{N}} \end{array}$$

Where N is the total number of detected categories.

(4) mAP50-95: The mAP corresponding to each IoU threshold is calculated and averaged over a range of intersection-over-union (IoU) thresholds from 0.5 to 0.95 (in steps of 0.05) and is used to comprehensively evaluate the robustness of the model under different localization accuracy requirements.

In the target detection system, mAP50–95 is the most comprehensive metric because it covers multiple IoU thresholds and is prioritized as the core index. mAP, precision, and recall are used as auxiliary analytical bases in turn. This design avoids the evaluation bias that may be introduced by a single IoU threshold (e.g., mAP50).

### 4.2 YOLOv12 baseline model performance evaluation

This section aims to scientifically identify the optimization space of the YOLOv12 model in litchi variety detection. To this end, the following core objectives are set:

• To establish the baseline performance, quantify the basic performance of the model, and provide a comparative baseline for optimization.

• To identify the key bottlenecks and locate the model’s deficiencies in lightweighting and similar-variety differentiation.

• To guide the direction of improvement and clarify the technical path for subsequent optimization.

Based on the 12-litchi dataset, this chapter conducts a comprehensive evaluation of its lightweight version, YOLOv12, to achieve the above objectives.

Under the standard test environment, 1,469 images covering 12 Litchi species are used as the test set, and the NVIDIA RTX 3060 GPU hardware platform is used to evaluate the comprehensive performance of the YOLOv12 model.

#### 4.2.1 YOLOv12 baseline overall performance analysis

The YOLOv12 baseline model demonstrates robust overall performance on the litchi variety detection task. The comprehensive detection metrics, presented in **Table 3**, establish a reliable baseline for subsequent optimization efforts. The overall detection indexes show that the model precision (P) reaches 93.4%, indicating that the false detection rate is at a low level; the mAP50 reaches 96.9%, which is excellent under the loose localization criterion; and the mAP50–95 reaches 93.6%, which is slightly lower than the mAP50 but still reflects a strong comprehensive detection capability.

**Table 3:** YOLOv12 baseline overall performance.

Indicator	YOLOv12
Precision P	0.934
Recall R	0.921
mAP50	0.969
mAP50-95	0.936
Number of parameters	2570388
Calculated Volume	6.5G

However, the model still has room for optimization in key dimensions. The recall (R) is 92.1%, which reveals certain missed detections, and the relatively lower mAP50–95 (93.6%) indicates that its generalization ability needs improvement under stringent localization requirements. In terms of model complexity, the parameter count of 2.57 million is relatively high for deployment on devices with limited memory resources, and the computational cost of 6.5 GFLOPs may lead to efficiency bottlenecks on resource-constrained hardware. These findings indicate that the current model still requires further optimization to improve its utility in lightweight deployments, such as mobile scenarios.

#### 4.2.2 YOLOv12 baseline analysis of detection performance for each species

Further analysis of per-variety detection performance reveals significant variation. As shown in **Table 4**, the mAP50 of conventional varieties such as Glu-Rice Ciba Litchi was as high as 99.5%, and the mAP50–95 of varieties such as Mar-Orange Litchi and Concubine-Smile Litchi exceeded 98%, indicating strong baseline performance for most varieties. The average precision for the remaining varieties exceeded 95%, and the average recall exceeded 92%, further validating the reliability of YOLOv12 for conventional variety detection. (Note: Model parameter count *=* 2,570,388; computational cost *=* 6.5 GFLOPs*.)*.

**Table 4:** Comparison of YOLOv12’s performance on detection of varieties.

Model|Performance	P	R	mAP50	mAP50-95
YOLOv12	0.934	0.921	0.969	0.936
Osmanthus-Fragr Litchi	**0.574**	0.995	0.889	0.849
Glu-Rice Ciba Litchi	0.999	1	0.995	0.959
Trib-Royal Litchi	0.984	0.983	0.994	0.935
Chk-Beak Litchi	0.95	0.995	0.995	0.947
Icy-Flesh Litchi	0.942	**0.492**	0.86	0.837
Wax-Shiny Leaf Litchi	0.961	0.979	0.99	0.963
Grn-Circle Litchi	1	0.801	0.977	0.915
Mar-Orange Litchi	0.959	0.959	0.993	0.983
Honey-Jar Litchi	0.957	0.915	0.973	0.913
Concubine-Smile Litchi	0.99	0.975	0.99	0.975
Blk-Green Leaf Litchi	0.92	0.923	0.973	0.967
Sci-Cultivar No.1	0.973	1	0.995	0.989

Bold values indicate the data values that require special attention.

**Figure 8:**
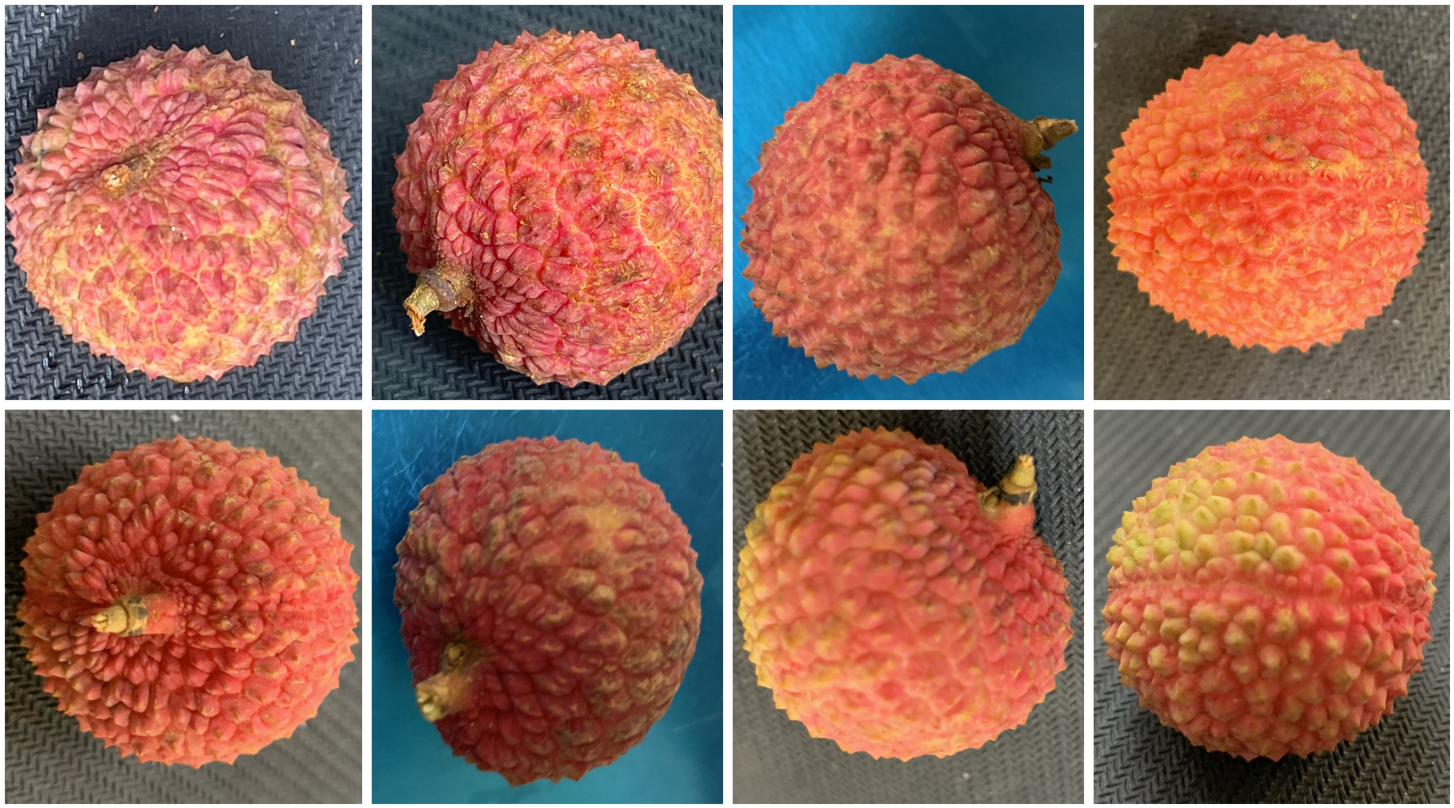
Easily confused Icy-Flesh Litchi and Osmanthus-Fragr Litchi samples.

However, significant challenges remain for specific varieties: the recall rate for *Icy*-Flesh *Litchi* was notably low at 49.2%, indicating that over half of the true instances were missed. This primarily stems from the loss of its pericarp’s micro-concave texture information in deeper feature maps. Conversely, the precision for *Osmanthus*-Fragr *Litchi* was only 57.4%, largely attributable to the misclassification of a substantial number of *Icy*-Flesh *Litchi* samples (46.2% misclassification rate). The similar color distribution between these two varieties and interference from branch and leaf shadows in the background further exacerbated the classification confusion. Grn-Circle Litchi, as a long-tailed variety, had a recall rate of 80.1%, reflecting the impact of uneven data distribution. These issues provide a clear direction for subsequent targeted optimization. Visual examples of these two challenging varieties are provided in **Figure 8** for reference.

### 4.3 YOLO-LitchiVar model performance

#### 4.3.1 Lightweighting effect and overall performance

When the DSC3k2, C2PSA, and ECA modules are integrated simultaneously, the model achieves optimal performance in the key metrics of precision (P = 93.4%), recall (R = 93.5%), mAP50 (97.7%), and mAP50–95 (94.4%). The number of parameters (approximately 2.2 M) and computation volume (5.6 G) remain at low levels. The experimental results are shown in **Table 5**. Although other module combinations exhibit slight differences in some indicators, the overall results show that multi-module co-optimization effectively improves recognition accuracy while meeting the lightweight requirements of the model. **Bold** values indicate optimal or suboptimal indicators. The unit for parameter quantity represents the number of parameters.

**Table 5:** Comparison of the comprehensive performance of different model architectures on the task of Litchi variety identification.

Baseline	Model			P	R	mAP50	mAP50-95	Params	FLOPs
	DSC3k2	C2PSA	ECA						
YOLOv12				0.934	0.921	0.969	0.936	2570388	6.5G
	✓			0.91	0.926	0.965	0.931	2515876	5.9G
		✓		0.914	0.935	0.973	0.939	**2203100**	**5.6G**
			✓	0.932	0.923	0.973	0.94	2523038	6G
	✓	✓		0.925	0.916	0.966	0.931	2206116	**5.6G**
	✓		✓	0.92	0.93	0.97	0.936	2516910	5.9G
		✓	✓	0.905	0.927	0.96	0.928	2213262	5.7G
	✓	✓	✓	**0.934**	**0.935**	**0.977**	**0.944**	**2207150**	**5.6G**

Bold values indicate the best performance for each metric.

**Figure 9:**
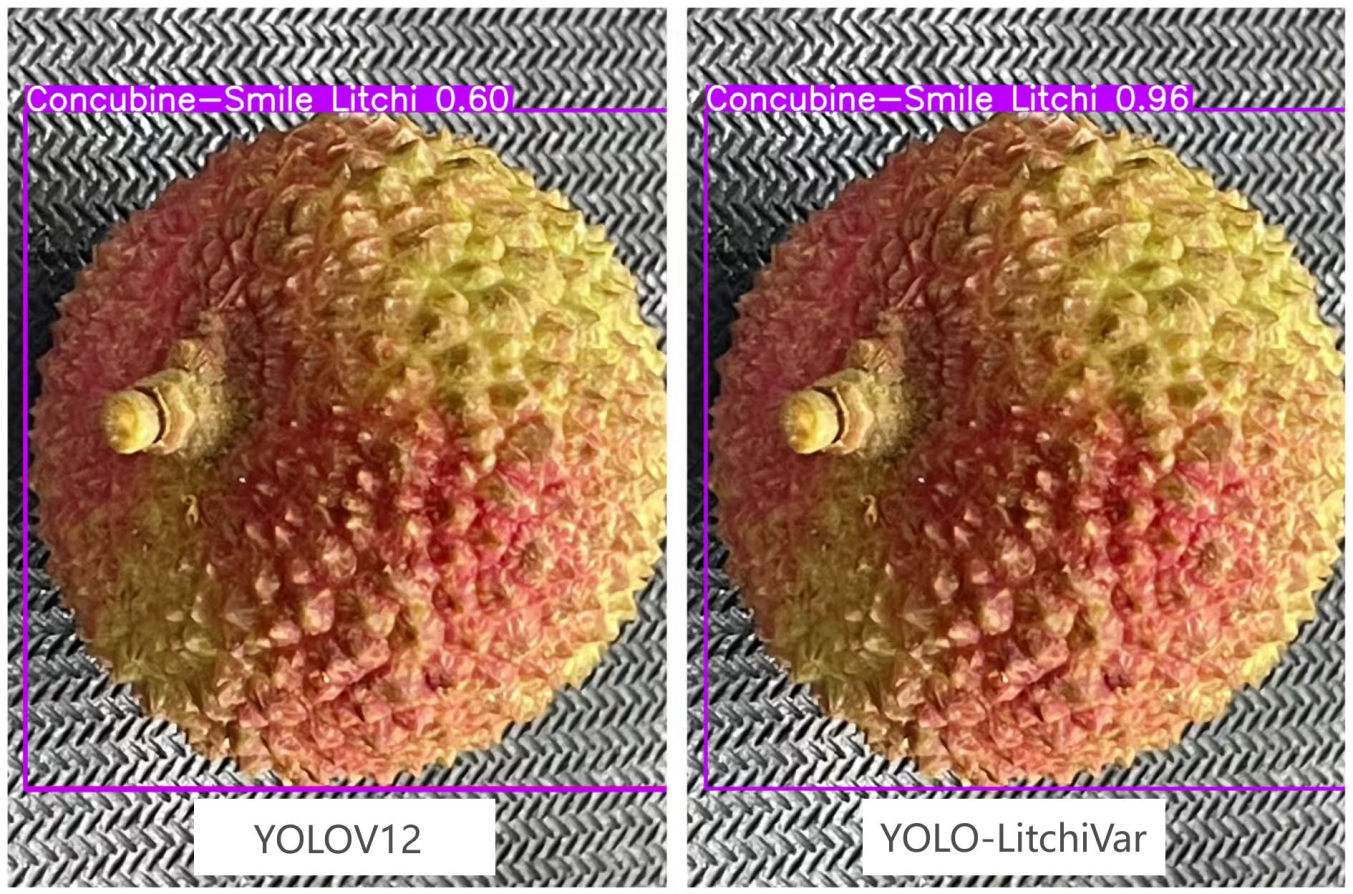
YOLOv12 and YOLO-LitchiVar model detection results.

As shown in **Figure 9**, the actual accuracy of the YOLO-LitchiVar model is higher than that of the YOLOv12 model on the litchi variety recognition task.

##### 4.3.1.1 Lightweight performance is reached

① DSC3k2 module contributes significantly: applying DSC3k2 alone compresses the number of parameters from 2.57 M to 2.52 M (a 5.4% reduction) and reduces the computational cost from 6.5 G to 5.9 G (a 9.2% reduction), verifying its efficient spatial–channel decoupling characteristics.

② C2PSA module combines performance and lightweighting: while C2PSA alone improves recall and mAP50-95, it also exhibits excellent lightweight characteristics, with a reference count of only 2.20M and a computational volume of 5.6G FLOPs.

③ Three-module synergistic optimization achieves the goal: the YOLO-LitchiVar model maintains or even exceeds the accuracy of the baseline model, with a precision (P) of 0.934 and a 1.4% increase in recall (R). The model achieves comprehensive efficiency improvements: parameter count is reduced from 2,570,388 to 2,207,150 (a 14.1% reduction), computational cost decreases from 6.5 G to 5.6 G FLOPs (a 13.8% reduction), and actual model size decreases from 5.3 MB to 4.5 MB (a 15.1% reduction). This multi-dimensional lightweighting fully meets the stringent resource constraints of mobile and embedded device deployment, addressing both computational and storage limitations.

##### 4.3.1.2 Overall accuracy improvement

The ablation study reveals distinct contributions of each module. Introducing the C2PSA module alone significantly improved recall from 0.921 to 0.935 (a 1.4% increase), demonstrating its effectiveness in capturing fine-grained microtextural features and addressing missed detection issues, particularly for challenging varieties such as *Icy*-Flesh *Litchi*. The ECA module, when introduced independently, showed a more modest improvement in recall (from 0.921 to 0.923) but achieved the highest mAP50–95 improvement among single-module additions (from 0.936 to 0.940, a 0.4% increase), indicating its superiority in enhancing precise localization and classification confidence through channel-wise feature recalibration. The DSC3k2 module primarily contributed to model efficiency, reducing parameters by 5.4% and computation by 9.2% while maintaining comparable accuracy. The synergistic combination of all three modules achieved the optimal balance, simultaneously enhancing detection accuracy (mAP50–95: 0.944) and model efficiency (Params: 2.2 M, FLOPs: 5.6 G).

### 4.4 Optimization of key variety identification

Among the 12 litchi varieties analyzed, *Icy*-Flesh *Litchi* and *Osmanthus*-Fragr *Litchi* exhibit highly similar pericarp texture and color characteristics. Both varieties display fine, irregularly arranged areoles with only minimal differences in density, edge sharpness, and groove depth, making them difficult to distinguish using traditional texture analysis algorithms. The color is dominated by reddish-purple tones, with subtle differences in shade, which further contributes to confusion due to color similarity. In addition, the tiny waxy particles on the pericarp surface of both varieties produce high light reflection, increasing the difficulty of distinguishing them by gloss or texture details. The detection results of the related models on these two varieties are shown in **Table 6**.

**Table 6:** Comprehensive performance comparison of different models on the identification task of the key similar varieties Icy-Flesh Litchi and Osmanthus-Fragr Litchi.

Model configuration	Icy-Flesh Litchi			Osmanthus-Fragr Litchi		
	P	R	mAP50	P	R	mAP50
Baseline (YOLOv12)	0.942	0.492	0.86	0.574	0.995	0.889
+ DSC3k2	0.972	0.599	0.895	0.603	0.995	0.913
+ ECA	0.97	0.557	0.915	0.595	0.99	0.907
+ C2PSA	0.952	0.599	0.924	0.558	0.979	0.899
+ DSC3k2 + ECA	0.966	0.61	0.938	0.602	0.995	0.896
+ DSC3k2 + C2PSA	0.929	0.568	0.903	0.603	0.985	0.9
+ C2PSA + ECA	0.898	0.487	0.835	0.542	1	0.892
+ DSC3k2 + C2PSA + ECA	**0.976**	**0.706**	**0.955**	**0.665**	**0.99**	**0.966**

Bold values indicate the best performance for each metric.

The ablation study reveals distinct contributions of each module. Introducing the C2PSA module alone significantly improved the recall value from 0.921 to 0.935 (a 1.4% increase), demonstrating its effectiveness in capturing fine-grained microtextural features and addressing missed detection issues, particularly for challenging varieties such as *Icy*-Flesh *Litchi*. The ECA module, when introduced independently, showed a more modest improvement in recall (from 0.921 to 0.923) but achieved the highest mAP50–95 improvement among single-module additions (from 0.936 to 0.940, a 0.4% increase), indicating its superiority in enhancing precise localization and classification confidence through channel-wise feature recalibration. The DSC3k2 module primarily contributed to model efficiency, reducing parameters by 5.4% and computation by 9.2% while maintaining comparable accuracy. The synergistic combination of all three modules achieved the optimal balance, simultaneously enhancing detection accuracy (mAP50–95: 0.944) and model efficiency (Params: 2.2M, FLOPs: 5.6G).

The experimental results also show the impact of different module combinations (DSC3k2, C2PSA, ECA) on the performance of the YOLOv12 model in recognizing key similar litchi varieties—Icy-Flesh Litchi versus Osmanthus-Fragr Litchi. With the stacking of modules, the comprehensive model performance gradually improves. The YOLOv12 baseline achieved an accuracy of 0.942 in Icy-Flesh Litchi recognition but had a relatively low recall (0.492) and mAP50 (0.86). It showed better performance in Osmanthus-Fragr Litchi recognition, with a recall of 0.995 and mAP50 of 0.889. When the DSC3k2, C2PSA, and ECA modules were integrated simultaneously, the model achieved the highest accuracy (0.976), recall (0.706), and mAP50 (0.955) for Icy-Flesh Litchi, and mAP50 of 0.966 for Osmanthus-Fragr Litchi. These results indicate that multi-module synergistic optimization can significantly improve recognition accuracy for similar varieties, particularly enhancing differentiation under complex scenarios.

(1) Optimization of Icy-Flesh Litchi recognition

①Recall rate increases dramatically by 43.5%: from 0.492 to 0.706 relative to the baseline, indicating that the model’s ability to detect real Icy-Flesh Litchi samples has been revolutionized, and the number of missed samples has been reduced significantly. The module contribution path is clear:

-DSC3k2: the recall improves by 21.7% from 0.492 to 0.599, compressing the parameters while preserving key microtexture information.

-+C2PSA (DSC3k2+C2PSA): recall improves from 0.492 to 0.568, a cumulative improvement of 15.4%, cross-layer fusion significantly enhances micro-concave texture (LBP feature) characterization.

-+ECA (three modules): recall improves from 0.492 to 0.706, with a cumulative improvement of 43.5%, suppressing noisy channels caused by light and stalk shading while focusing on key microtexture channels.

②Accuracy and mAP50 increase simultaneously: accuracy reached 0.976, a 3.6% improvement over the baseline; and mAP50 reached 0.955, an 11.0% improvement from the baseline. This proves that the increase in recall was not at the expense of precision but achieved both high precision and high recall.

(2) Optimization of Osmanthus-Fragr Litchi recognition

①Precision improved by 15.8%: from 0.574 to 0.665, significantly reducing the probability of the model misclassifying other samples (especially Icy-Flesh Litchi and background) as Osmanthus-Fragr Litchi.

**Figure 10:**
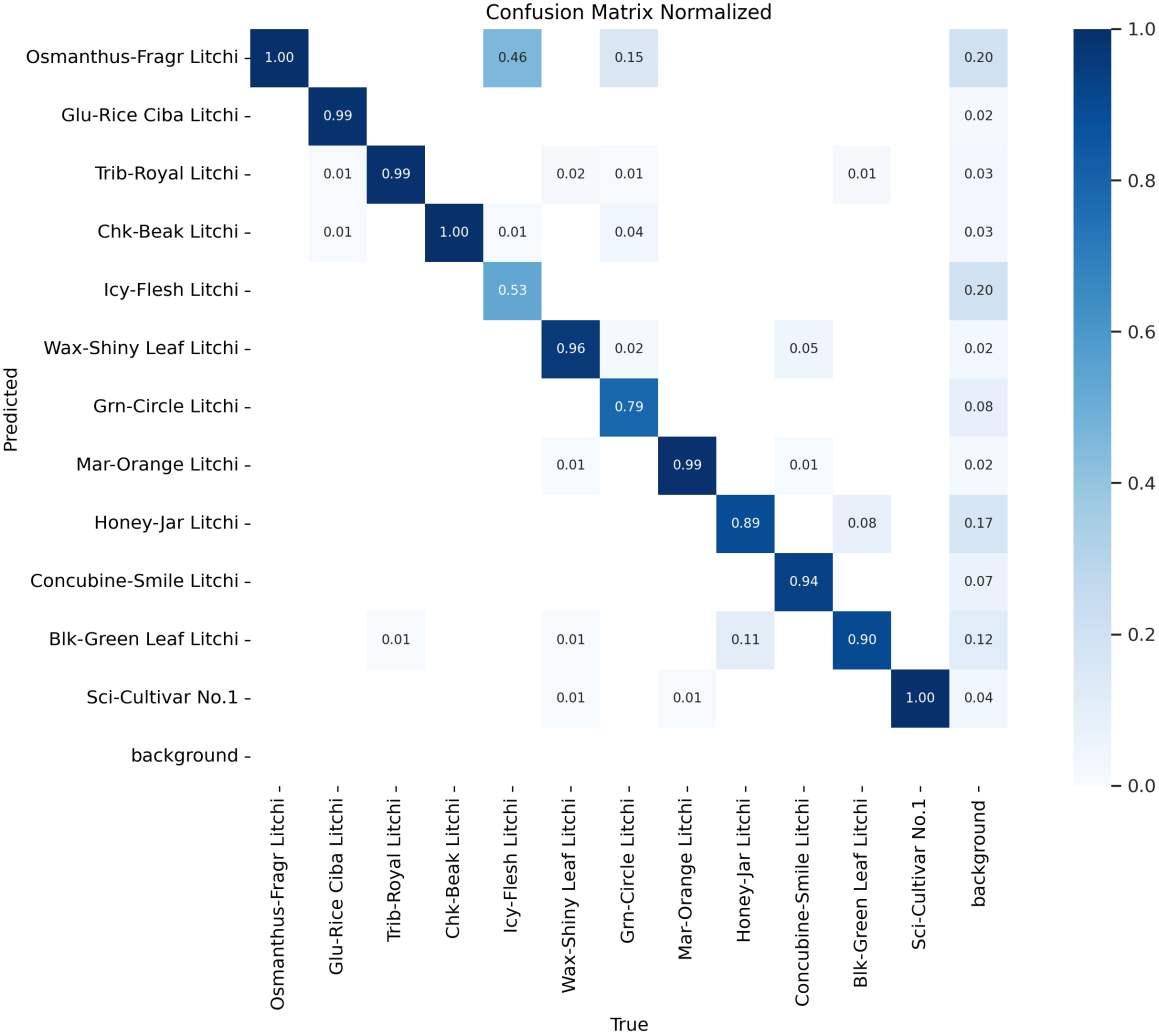
YOLOv12-confusion_matrix_normalized.

**Figure 11:**
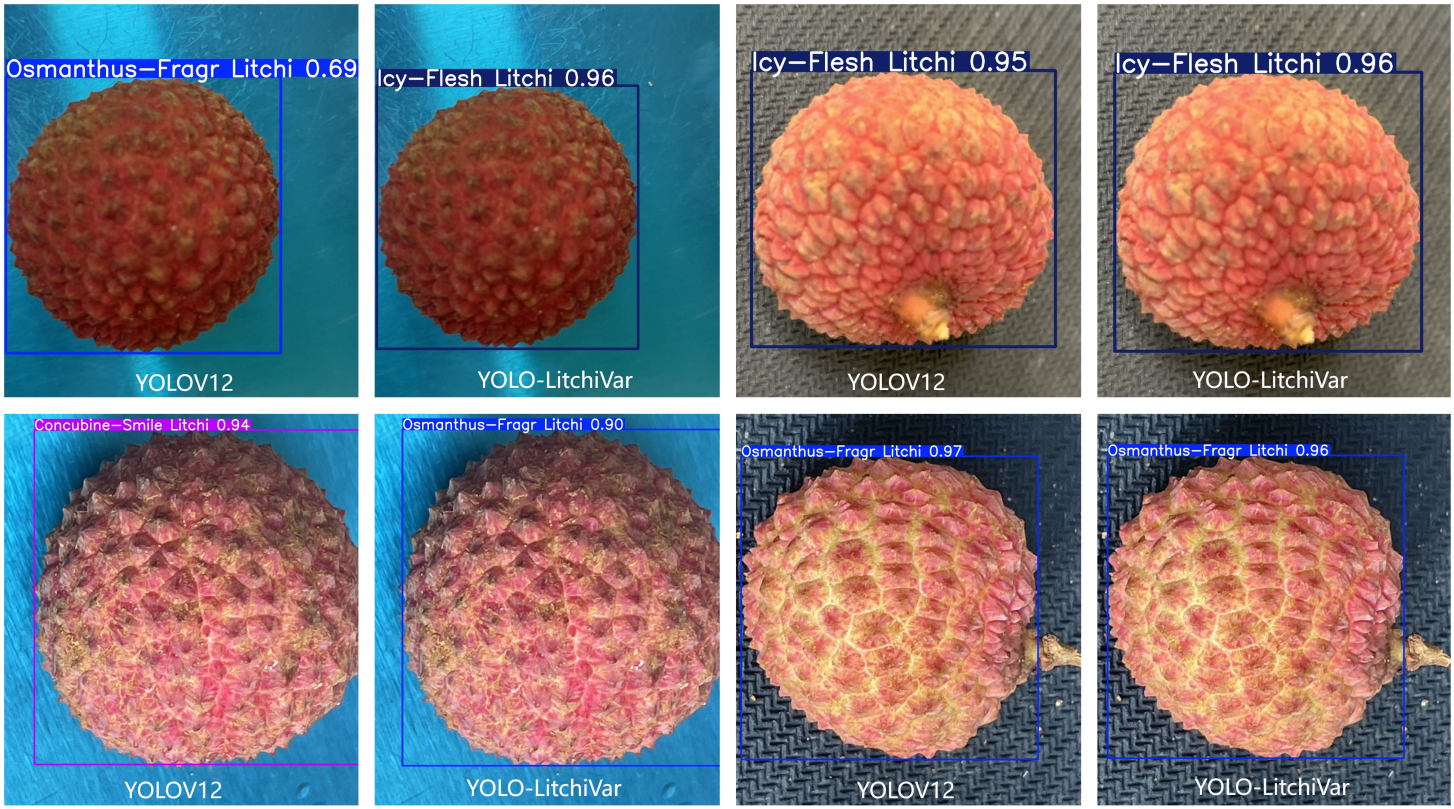
Detection of YOLOv12 and YOLO-LitchiVar models in confused varieties.

②Misclassification reduction by 19.1%: the probability of Icy-Flesh Litchi being misclassified as Osmanthus-Fragr Litchi is sharply reduced by 19.1%: the misclassification confusion matrices of the YOLOv12 and YOLO-LitchiVar models are shown in **Figures 10**, **11**, respectively, and the probability of Icy-Flesh Litchi being misclassified as Osmanthus-Fragr Litchi is reduced from 0.462 in the YOLOv12 baseline model to 0.34 in the YOLO-LitchiVar model. This improvement is mainly attributed to:

-C2PSA: enhanced feature differentiation between Icy-Flesh Litchi microtexture and Osmanthus-Fragr Litchi sharp cracked slices.

-ECA: dynamic adjustment of channel weights to suppress shared noise features such as high light reflection, reduce low-weight channel share enhancement, and highlight respective discriminative channels.

### 4.5 Ablation comparison results

From **Table 5** and **Table 6**, we can analyze and see that the ablation experiment and the key similar-variety comparison experiment fully verify the significant performance improvement of the YOLO-LitchiVar model and realize the synergistic optimization of lightweight design and key-variety differentiation accuracy. The schematic diagram of the detection comparison between the YOLOv12 and YOLO-LitchiVar models for confusing varieties is shown in **Figure 11**.

### 4.6 Analysis of training loss curves

**Figure 12:**
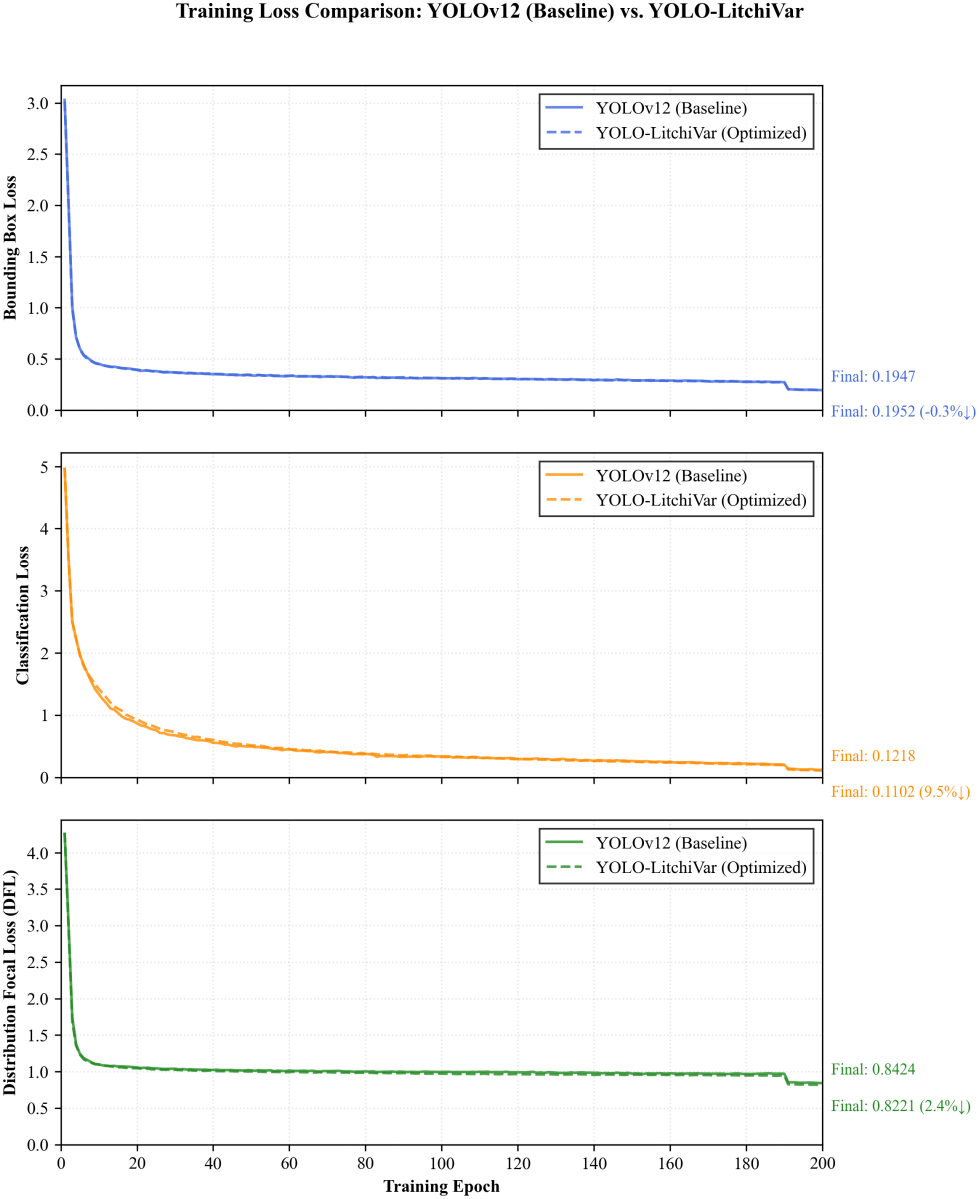
Training loss curves of the YOLO-LitchiVar model, showing the classification loss (cls_loss), bounding box regression loss (box_loss), and Distribution Focal Loss (dfl_loss) over 200 epochs.

To provide a comprehensive understanding of the training dynamics and convergence behavior of the proposed YOLO-LitchiVar model, the evolution of its primary loss components over 200 training epochs is analyzed. The training loss curves, illustrated in **Figure 12**, depict the progression of the classification loss (cls_loss), bounding box regression loss (box_loss), and the distribution focal loss (dfl_loss).

All three loss components exhibit a consistent and monotonic decreasing trend throughout the training process, ultimately converging to stable plateaus. This behavior signifies effective learning and optimization without signs of divergence or catastrophic overfitting. The box_loss, which penalizes inaccuracies in predicting the bounding box coordinates, decreases rapidly during the initial epochs and continues to refine throughout training, indicating the model’s progressive improvement in precisely localizing litchi fruits within the images.

The cls_loss, which measures the error in variety classification, also shows a steep decline initially, followed by a more gradual reduction. This pattern reflects the model’s successful learning of discriminative features necessary to distinguish between the 12 litchi varieties. The convergence of cls_loss to a very low value underscores the model’s high classification capability.

The dfl_loss, a component specific to the YOLO architecture that improves bounding box accuracy by modeling the distribution of box coordinates, demonstrates a similar descending trajectory. Its successful minimization confirms that the model not only predicts the presence and class of objects accurately but also precisely defines their spatial boundaries.

The synchronized descent and stabilization of all three loss curves validate the stability of the training process and the effectiveness of the chosen hyperparameters. The absence of significant oscillations or rebounds in the curves after the initial learning phase suggests that the model robustly learned generalizable features without overfitting to the training data. This analysis of the training loss curves provides crucial insights into the model’s learning process and complements the high-performance metrics reported on the independent test set, further confirming the reliability of the YOLO-LitchiVar model.

### 4.7 Comparative analysis with YOLO series models

**Figure 13:**
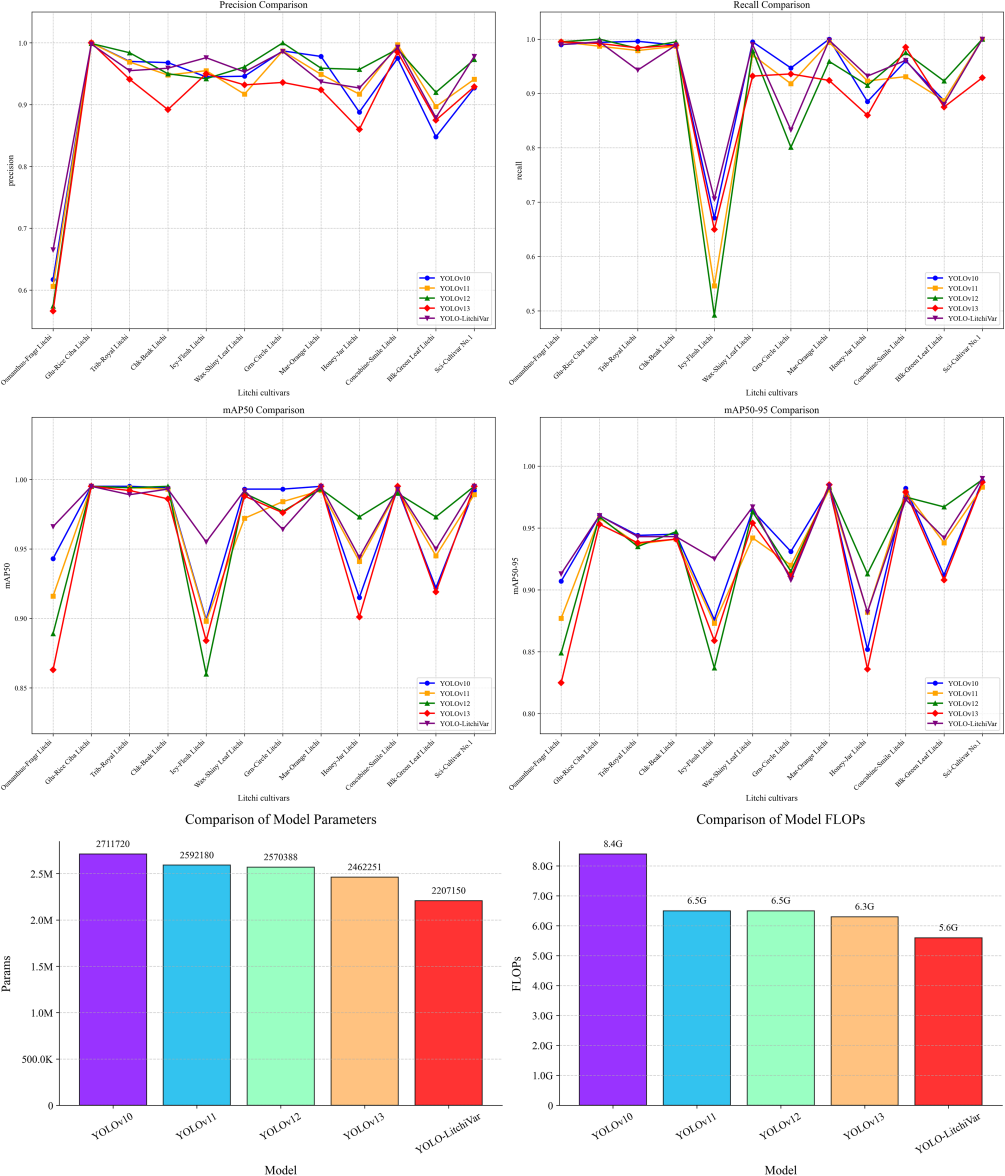
Performance comparison of YOLO-LitchiVar and YOLO models by version.

To comprehensively assess the sophistication of the YOLO-LitchiVar model in the task of litchi variety identification, a side-by-side comparison was conducted with four mainstream versions—YOLOv10, YOLOv11, YOLOv12, and YOLOv13. This focused comparison within the YOLO family ensures a consistent experimental framework and evaluation protocol, allowing for a direct assessment of the architectural improvements introduced by the proposed modules. The experiment used a unified dataset of 11,998 images with a 7:2:1 division consistent with the preceding experiments. Precision (P), recall (R), mean average precision (mAP50, mAP50*–*95), number of parameters (Params), and computation volume (FLOPs) were used as the core evaluation metrics. The comparative results are shown in **Table 7**, and the performance comparison between YOLO-LitchiVar and other YOLO versions is visualized in **Figure 13**.

**Table 7:** Comprehensive performance comparison between YOLO-LitchiVar and YOLO series models.

Model	P	R	mAP50	mAP50-95	Params	FLOPs	Model size
YOLOv10	0.921	0.943	0.969	0.937	2,711,720	8.4G	5.47MB
YOLOv11	0.924	0.927	0.968	0.934	2,592,180	6.5G	5.20MB
YOLOv12 (baseline)	0.934	0.921	0.969	0.936	2,570,388	6.5G	5.30MB
YOLOv13	0.899	0.936	0.957	0.923	2,462,251	6.3G	5,13MB
YOLO-LitchiVar	0.934	0.935	0.977	0.944	2,207,150	5.6G	4,50MB

The comparative analysis delineated in **Table 7** provides a compelling quantitative narrative of the YOLO-LitchiVar model’s performance. However, a mere presentation of metrics falls short of capturing the substantive architectural innovations driving these results. A deeper qualitative analysis reveals that the observed superiority stems from a targeted addressal of the specific challenges inherent to fine-grained agricultural object detection, particularly litchi variety discrimination.

YOLO-LitchiVar achieves the highest mAP50–95 (94.4%) and mAP50 (97.7%) among all compared versions, which are the most comprehensive metrics for evaluating localization accuracy and overall detection robustness. This superiority is further confirmed by its balanced and high performance in precision (0.934) and recall (0.935), indicating a significant reduction in both false positives and false negatives compared with the baseline and other variants.

More importantly, this performance breakthrough is achieved concurrently with substantial enhancement in model efficiency across multiple dimensions. With only 2.2 million parameters and 5.6 GFLOPs, YOLO-LitchiVar demonstrates superior computational efficiency. Crucially, the actual model size of 4.5 MB represents the most compact implementation among all compared YOLO variants, providing a practical advantage for storage-constrained deployment scenarios. This 15.1% reduction in model size compared with YOLOv12 (5.3 MB) and 17.7% reduction compared with YOLOv10 (5.47 MB) underscores the effectiveness of our architectural optimizations in achieving genuine lightweight characteristics without compromising accuracy.

This multi-dimensional efficiency achievement validates our core design philosophy: lightweighting is not merely about minimizing computational metrics in isolation but about optimizing the trade-off across parameters, computational complexity, and practical storage requirements to achieve the highest possible accuracy under constraints feasible for edge-device deployment. The model’s efficiency metrics are not just low—they are optimally balanced for achieving superior performance in resource-constrained environments. This leading performance can be directly attributed to the synergistic effect of the three proposed modules: the DSC3k2 module provides foundational parameter reduction; the C2PSA module effectively mitigates the information loss typically associated with model compression by enhancing shallow feature representation (evidenced by the drastic recall improvement for *Icy*-Flesh *Litchi*); and the ECA module refines the feature discrimination power, effectively resolving interclass confusion. The results show that our co-optimization strategy successfully breaks the inherent accuracy–efficiency trade-off that often constrains lightweight model design.

## 5 Conclusion

The accurate and efficient identification of litchi varieties post-harvest represents a significant bottleneck in the modernization of the litchi industry, directly impacting economic value realization and market fairness. Traditional manual methods are inherently subjective, inefficient, and non-scalable. To address this critical challenge, this study proposes YOLO-LitchiVar, a novel object detection model founded upon the YOLOv12 architecture but significantly enhanced through a synergistic triple-module co-optimization strategy specifically designed for the fine-grained task of litchi variety discrimination and the practical constraints of mobile deployment.

The contributions of this work are multifaceted and quantitatively validated through comprehensive experimentation:

Foundational lightweighting through architectural innovation: The introduction of the DSC3k2 module, which systematically replaces standard convolutional layers with a depthwise separable structure, serves as the cornerstone of model efficiency. This design decision—grounded in the decoupling of spatial filtering and channel fusion, as detailed in Section 3.2.2.1 and Equations 5–7—resulted in a 14.1% reduction in model parameters (from 2.57 million to 2.20 million) and a 13.8% reduction in computational complexity (from 6.5 GFLOPs to 5.6 GFLOPs). This achievement establishes YOLO-LitchiVar as the most lightweight model within the entire YOLO series for this task, thereby fulfilling a primary prerequisite for deployment on resource-constrained edge devices.

Enhanced fine-grained feature representation for overcoming detection bottlenecks: The development of the C2PSA cross-layer feature aggregation module directly addresses a key limitation of previous models—the loss of critical shallow feature information, such as the micro-concave textural patterns on the pericarp of ‘Icy-Flesh Litchi’. By implementing a principled mechanism for multi-scale feature alignment and fusion (Section 3.2.2.2, Equation 8), the C2PSA module enriches the feature maps provided to the detection head. The efficacy of this module is demonstrated by a 43.5% increase in recall (from 0.492 to 0.706) for the challenging *Icy*-Flesh *Litchi* variety (Section 4.4, **Table 6**), effectively mitigating previously high missed-detection rates and contributing to a 1.4% improvement in the model’s overall recall (R).

**Figure 14:**
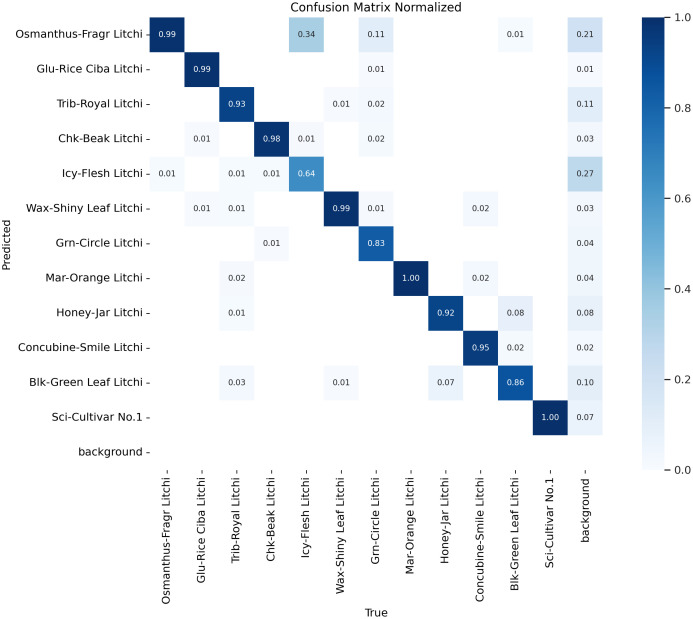
YOLO-LitchiVar-confusion_matrix_normalized.

Superior discriminative power for resolving interclass similarity: The integration of the ECA efficient channel attention mechanism provides dynamic feature refinement capability. By performing adaptive channel-wise calibration (Section 3.2.2.3, Equations 9–13), this module selectively amplifies features that are discriminative for specific varieties while suppressing semantically redundant features and background noise (e.g., lighting variations, leaf shadows) common across varieties. This capability is crucial for distinguishing morphologically similar varieties such as *Icy*-Flesh *Litchi* and *Osmanthus*-Fragr *Litchi*. The result is a 19.1% reduction in their mutual misclassification rate (from 0.462 to 0.340) and a 15.8% improvement in the precision of *Osmanthus*-Fragr *Litchi* (from 0.574 to 0.665), as analyzed in Section 4.4 and visualized in the confusion matrices (**Figures 10**, **14**).

The synergistic integration of these three modules enables the YOLO-LitchiVar model to achieve new state-of-the-art performance on the constructed litchi variety dataset. The model attains a mAP50–95 of 94.4% and a mAP50 of 97.7%, surpassing all mainstream YOLO versions (YOLOv10, v11, v12, v13) in overall accuracy, as compared in Section 4.7 and **Table 7**. Notably, this performance excellence is achieved while maintaining the smallest model size (4.5 MB) among all variants, representing a 15.1% reduction from the baseline YOLOv12 model. This optimal balance between accuracy and lightweight characteristics across multiple efficiency metrics (parameters, FLOPs, and model size) underscores the model’s practicality and readiness for real-world, in-field application in storage-constrained environments.

Despite these advancements, certain limitations highlight directions for future work. The current study was conducted under controlled laboratory conditions, which may limit immediate applicability to variable field environments. Future research should focus on:

1. validating model performance in outdoor settings with natural illumination and complex backgrounds;
2. conducting comprehensive cross-architecture benchmarking with other lightweight detectors beyond the YOLO series;
3. enhancing robustness for varieties with long-tailed data distributions (e.g., *Grn*-Circle *Litchi*) through advanced data augmentation and class-imbalance learning techniques; and
4. integrating spatial attention mechanisms and morphological priors (e.g., geometric properties of cracked patches) to further improve discrimination between visually similar varieties under challenging field conditions.

## Author’s note

Bing Xu received the Ph.D. degree from the Nanjing Normal University. He has been engaged in research on wireless Internet of Things (IoT) and image processing. He is a professor and the Supervisor for Master’s at Guangdong University of Petrochemical Technology, Guangdong. Xianjun Wu received the Master’s degree in software engineering from the Huazhong University of Science and Technology, in 2006. Now, he is engaged in computer and image processing research at Guangdong University of Petrochemical Technology, Guangdong. Xueping Su undergraduate student. She is currently studying at School of Computer, Guangdong University of Petrochemical Technology, Guangdong Province, China. Wende Ke, received a Ph.D. degree in Automatic Control from Harbin Institute of Technology, Department of Mechanical and Energy Engineering, Southern University of Science and Technology, Shenzhen, 518055, China.

## Data Availability

The datasets presented in this study can be found in online repositories. The names of the repository/repositories and accession number(s) can be found below: https://doi.org/10.57760/sciencedb.28666.
